# Differential regulation of the immune system in a *brain-liver-fats* organ network during short-term fasting

**DOI:** 10.1016/j.molmet.2020.101038

**Published:** 2020-06-08

**Authors:** Susie S.Y. Huang, Melanie Makhlouf, Eman H. AbouMoussa, Mayra L. Ruiz Tejada Segura, Lisa S. Mathew, Kun Wang, Man C. Leung, Damien Chaussabel, Darren W. Logan, Antonio Scialdone, Mathieu Garand, Luis R. Saraiva

**Affiliations:** 1 Sidra Medicine, PO Box 26999, Doha, Qatar; 2 Institute of Epigenetics and Stem Cells, Helmholtz Zentrum München, Marchioninistraße 25, 81377, München, Germany; 3 Institute of Functional Epigenetics, Helmholtz Zentrum München, Ingolstädter Landstraße 1, 85764, Neuherberg, Germany; 4 Institute of Computational Biology, Helmholtz Zentrum München, Ingolstädter Landstraße 1, 85764, Neuherberg, Germany; 5 Wellcome Sanger Institute, Wellcome Genome Campus, Hinxton, Cambridge, CB10 1SD, UK; 6 Monell Chemical Senses Center, 3500 Market Street, Philadelphia, PA, 19104, USA

**Keywords:** Fasting, Multiorgan, RNA-seq, Immune system, Systems biology

## Abstract

**Objective:**

Fasting regimens can promote health, mitigate chronic immunological disorders, and improve age-related pathophysiological parameters in animals and humans. Several ongoing clinical trials are using fasting as a potential therapy for various conditions. Fasting alters metabolism by acting as a reset for energy homeostasis, but the molecular mechanisms underlying the beneficial effects of short-term fasting (STF) are not well understood, particularly at the systems or multiorgan level.

**Methods:**

We performed RNA-sequencing in nine organs from mice fed *ad libitum* (0 h) or subjected to fasting five times (2–22 h). We applied a combination of multivariate analysis, differential expression analysis, gene ontology, and network analysis for an in-depth understanding of the multiorgan transcriptome. We used literature mining solutions, LitLab™ and Gene Retriever™, to identify the biological and biochemical terms significantly associated with our experimental gene set, which provided additional support and meaning to the experimentally derived gene and inferred protein data.

**Results:**

We cataloged the transcriptional dynamics within and between organs during STF and discovered differential temporal effects of STF among organs. Using gene ontology enrichment analysis, we identified an organ network sharing 37 common biological pathways perturbed by STF. This network incorporates the brain, liver, interscapular brown adipose tissue, and posterior-subcutaneous white adipose tissue; hence, we named it the *brain-liver-fats* organ network. Using Reactome pathways analysis, we identified the immune system, dominated by T cell regulation processes, as a central and prominent target of systemic modulations during STF in this organ network. The changes we identified in specific immune components point to the priming of adaptive immunity and parallel the fine-tuning of innate immune signaling.

**Conclusions:**

Our study provides a comprehensive multiorgan transcriptomic profiling of mice subjected to multiple periods of STF and provides new insights into the molecular modulators involved in the systemic immunotranscriptomic changes that occur during short-term energy loss.

## Highlights

• A multi-organ transcriptomic atlas of short-term fasting (STF) in mice.
• Data-driven grouping and gene functional clustering enhanced analytical depth.
• The shared biological processes in mice forms a brain-liver-fats network.
• STF orchestrates major changes in immune-related processes across many organs.

## Abbreviations

STF: Short-term fasting
OB: olfactory bulb
BRN: brain
CBL: cerebellum
BST: brainstem
STM: stomach
LIV: liver
iBAT: interscapular brown adipose tissue
pgWAT: perigonadal white adipose tissue
psWAT: posterior-subcutaneous white adipose tissue
HVG: highly variable genes
log2(x+1) NC: normalized log2 expression values
HCA: hierarchical clustering analysis
FDR: false discovery rate
DEGs: differentially expressed genes
FC: fold change
log2FC: log2 fold change
GO: Gene Ontology
subcDEGs: subclustered DEGs
LitLab™: Acumenta Literature Lab
MeSH: medical subject headings
GH: growth hormone
IGF: insulin-like growth factors
BDNF: brain-derived neurotropic factor
NE: norepinephrine
MGI: Mouse Genome Informatics
CR: caloric restriction

## 1 Introduction

Dietary restriction refers to an intervention that ranges from a chronic but minor reduction in calorie intake to periods of repeated cycles of short-term fasting (STF) and, in humans, fasts lasting more than 48 h [1]. These various forms of reduction in food consumption have many beneficial effects on health, including weight reduction, amelioration of autoimmune diseases, and increased lifespan [2,3]. In line with this, a triad of recent studies demonstrated the ability of different forms of fasting to alter the levels and functions of the various immune cell types [[4], [5], [6]]. The interest and applicability of utilizing fasting to treat various conditions are as such that they have recently generated momentum for clinical trials [[7], [8], [9]].

The advent of high-throughput omics facilitated the elucidation of some of the cellular and molecular mechanisms underlying the beneficial effects of fasting. Nonetheless, the majority of these studies have focused on single organ response (i.e., the liver) and have less often involved two or three organs [[10], [11], [12], [13], [14], [15], [16]]. The adaptation to energy deprivation, however, requires a multiorgan integration of metabolic modulations to protect the organism from an irreversible loss of resources [17]. In mice, disturbance of normal eating patterns alters metabolism systemically [18]. A recent study described the interorgan coordination of adaptive responses to various periods of fasting (0–72 h) in mice; however, the transcriptomic changes characterized in the four tissues applied mainly to states of starvation and are limited by the depth offered by microarrays [19]. Another recent multiomics approach also presented similar limitations [20]. Thus, despite this progress, a multiorgan or systemic study of the transcriptional dynamics underlying fasting has not been performed.

In this study, we profiled transcriptomic responses to periods of STF (2–22 h) in nine mouse organs. Using a combination of data-driven and semantic similarity clustering approaches, we discovered the presence of a *brain-liver-fats* organ network, conserved in their enriched biological processes perturbed by STF. The organ network recapitulated numerous reported physiological/molecular changes associated with various forms of fasting. In summary, we provide a multiorgan atlas of the known and novel molecular mediators of the systemic effects of short-term energy loss in mice.

## 2 Material and methods

### 2.1 Mice and STF experiments

**Figure 1:**
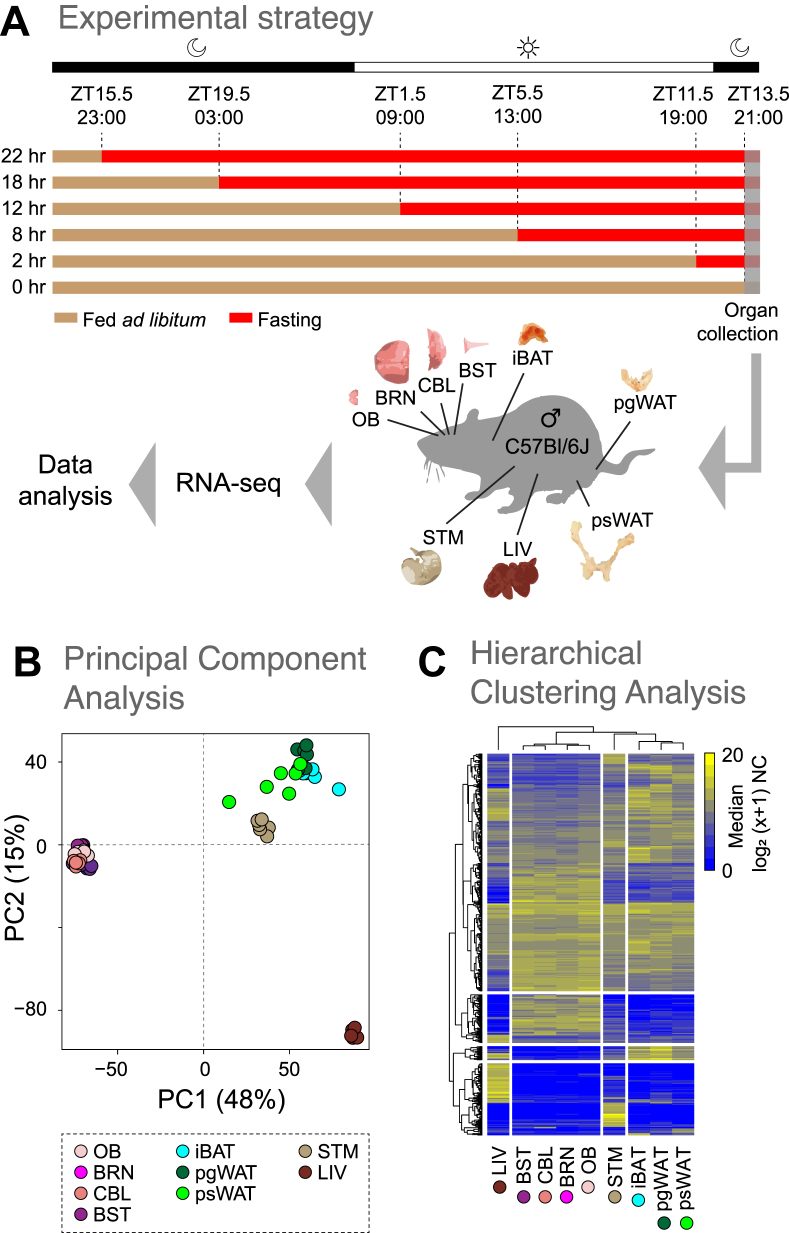
**Transcriptomic profiling of multiple organs in the fasted mice.A)** Schematic view of the experimental design for the five different short-term fasting (STF) durations. The fasting starting times are indicated by the red bars (0, 2, 8, 12, 18, and 22 h), along with the corresponding Zeitgeber times (ZT). The fed *ad libitum* condition is shown by the beige bars. The time of organ collection from male C57Bl/6J mice (n = 3 per time point) is represented by the grey-shaded area. Periods of light-on and light-off are represented by the white and black bars, respectively. **B)** Principal component (PC) analysis of the 9420 expressed genes in all samples. Each dot represents the gene expression profile of an organ (indicated by the color) at a specific time point. Percentages of the variance explained by the PCs are indicated in parentheses. **C)** Hierarchical clustering analysis on the union of the top 100 highly expressed genes (rows) among all organs (columns). Median mRNA expression levels are represented on a log_2_ (x+1) scale of normalized counts (NC) (0 - not expressed; 20 - highly expressed). Organ abbreviations: OB - olfactory bulb, BRN - brain, CBL - cerebellum, BST - brainstem, STM - stomach, LIV - liver, iBAT - interscapular brown adipose tissue, pgWAT - perigonadal white adipose tissue, and psWAT - posterior-subcutaneous white adipose tissue.

All animals used in this study were adult (8–9 weeks of age) C57Bl/6J males, group-housed since birth at the animal facility of the Wellcome Sanger Institute. Mice were kept on a 12:12 h light:dark schedule, with lights on at 07:30 (zeitgeber time 0, or ZT0). For the STF experiment, mice (n = 3 per time point) fasted for 2 (ZT11.5), 8 (ZT5.5), 12 (ZT1.5), 18 (ZT19.5), or 22 h (ZT15.5), and the control group (i.e., 0 h or ZT13.5) was fed *ad libitum* (Figure 1A). All mice had access to water throughout the assay. All animals were sacrificed by cervical dislocation after the start of the dark cycle (between 21:00 and 21:30, or ZT13.5–14). The following nine organs were collected and processed for mRNA-seq: olfactory bulb (OB), brain (BRN, which includes the telencephalon and diencephalon), cerebellum (CBL), brainstem (BST, which comprises the mesencephalon, pons, and myelencephalon), stomach (STM), liver (LIV), interscapular brown adipose tissue (iBAT), perigonadal white adipose tissue (pgWAT), and posterior-subcutaneous white adipose tissue (psWAT). The organs were immediately frozen and kept at −80 °C until further processing.

### 2.2 RNA extraction and RNA-sequencing

Organs (OB, BRN, CBL, BST, STM, LIV, and iBAT) were homogenized in Lysis RLT Buffer (Qiagen) supplemented with 1% β-mercaptoethanol (Sigma–Aldrich) by using the OMNI tissue homogenizer (OMNI International). Homogenized organ lysates were then loaded onto the QIAshredder homogenizer spin columns (Qiagen) for further homogenization and elimination of insoluble debris. Total RNA was extracted by using the RNeasy Mini Kit (Qiagen), according to the manufacturer's protocol. White adipose tissues (pgWAT and psWAT) were homogenized in Qiazol (Qiagen) and RNA extracted with the Lipid RNeasy Lipid Tissue Mini Kit (Qiagen). mRNA was prepared for sequencing by using the TruSeq stranded mRNA sample preparation kit (Illumina), with a selected insert size of 120–210 bp. All samples were sequenced on an Illumina HiSeq 4000, generating paired-end 150 bp sequencing reads, and had an average depth of 39.98 ± 1.05 (SEM) million reads (Additional file 2: Table S1).

### 2.3 Short reads alignment to reference genome and transcriptome

The quality of the reads was assessed by using FastQC (KBase). Sample Fastq files were aligned to the mouse reference genome mm10/GRCm38.p5 by using TopHat2 [21] with two mismatches allowed. Reads were retained only if uniquely mapped to the genome. We used HTSeq-count (0.9.1, -t exon and –m union) to obtain the number of reads mapped to each gene in Gencode M16. Bigwig files were generated from bam files for visualization by using RSeQC [22].

### 2.4 Pre-processing

The schematic of the bioinformatic workflow is presented in Additional file 1: Fig. S1. Before the analysis, the mapped read counts were filtered for annotated genes by using org.Mm.eg.db [23]. A count of a per million threshold equivalent to ∼10 raw expression value was applied to remove all lowly expressed genes, and only genes having ≥ 3 samples above the threshold were kept. Samples with total reads lower than two standard deviations from the organ means were removed.

### 2.5 Clustering group calls and bootstrap validations for the fasting phases

Highly variable genes (HVGs) were queried from normalized log_2_ expression values (log_2_ (x+1) NC) of the preprocessed datasets as informative genes and were used for the data-driven clustering calls. Briefly, we calculated the gene-specific variance and regressed against its mean log-expression value and applied a false discovery rate (FDR) < 0.05 to denote significance, which resulted in between 824 and 1190 HVGs per organ. Spearman's rho was then used to calculate the correlation distance matrix among these genes in each organ:

$$dist=sqrt\left(0.5*\left(1-Spearman^{'}s\;Rho\right)\right)$$

To determine the optimal number of clusters, hierarchical clustering (method = complete) was performed on the correlation distance matrixes of the HVGs and bootstrapped to 5000 iterations. A bootstrap mean cutoff (≥0.65) was used to determine the significance of the cluster fit, and any clusters not fulfilling the criteria were grouped with the previous node. A cluster size of three was found to be optimal for most organs, which we designated as fasting Phases I, II, and III (Additional file 3: Table S2).

### 2.6 Determining the differentially expressed genes (DEGs)

**Figure 2:**
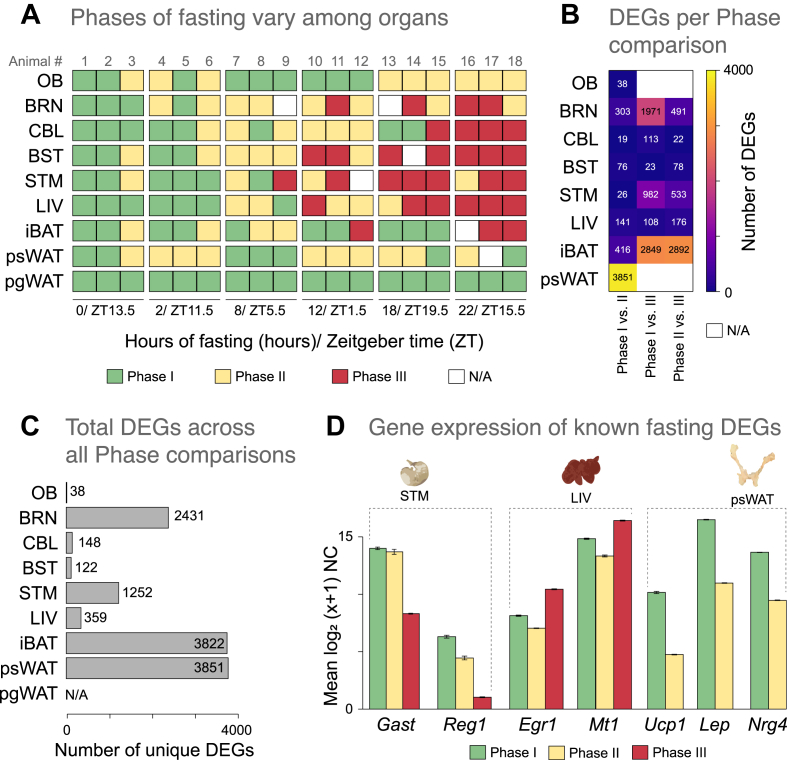
**Gene expression-driven sample clustering shows differential temporal effects of STF among organs.A)** Fasting Phase-call results from the data-driven clustering approach for each animal (columns) and organs (rows): Phase I (green), Phase II (yellow), Phase III (red). N/A (white) represents excluded samples that did not meet the QA/QC criteria (see Additional file 3: Table S2). Experimental durations of fasting are as indicated. **B)** Number of the differentially expressed genes (DEGs) from the all pairwise fasting Phase comparisons in all organs (see Methods). Because samples from pgWAT did not split into fasting Phases, no DEGs for the organ were available (N/A). **C)** Number of unique differentially expressed genes (DEGs) from the all pairwise fasting Phase comparisons in all organs (see Methods). Because samples from pgWAT did not split into fasting Phases, no DEGs for the organ were available (N/A). **D)** Expression values across the three Phases of select DEGs that have been previously reported to be modulated by fasting in select organs. Mean mRNA expression levels are represented on a log_2_ (x+1) scale of normalized counts (NC) ± SEM (for replicate number, see Additional file 3: Table S2). Organ acronyms are as in Figure 1.

The DEseq2 package was used to determine DEGs for all possible pairwise comparisons within each organ. We used two methods to determine DEGs: a pairwise manner between each time point against zero (data not shown), and a pairwise manner between the aforementioned Phases. The samples populating each Phase in each organ are summarized in Figure 2A. Genes were considered differentially expressed if they had a log_2_FC > 1.0 and FDR <0.05. All DEGs for the Phase comparison are provided in Additional file 4: Table S3—the columns contain the following data: *baseMean* corresponds to the mean normalized expression value for the gene across all samples; *log2FoldChange* is the fold change (FC) between the two groups tested, log2 transformed; *lfcSE* corresponds to the standard error associated with the FC estimation; *stat* is the Wald statistic; *p value* is the p value of the test; and *padj* is the p value after adjusting for multiple testing (Benjamini-Hochberg). For organs yielding >500 DEGs across all possible pairwise Phase comparisons, we kept only the top and bottom 12.5% ranked DEGs (filtered DEGs) to focus our downstream analyses on the genes displaying the highest variation (Fig. S4b).

### 2.7 Gene ontology (GO) enrichment analysis

Several strategies were used to perform the gene functional enrichment analysis to maximize annotation coverage and to provide meaningful interpretations for the DEGs. Briefly, the filtered DEGs (see above) from each organ were submitted, as a single gene set, to query for *Mus musculus* GO terms associated with biological processes by using the following algorithms and databases: the gene over-representation test (enrichGO) and gene set enrichment analysis, both of which are provided by ClusterProfiler [24], and the Database for Annotation, Visualization, and Integrated Discovery (DAVID, [25]). The minimal gene set size was set at three, and an FDR<0.05 cutoff was set for significance. When applicable, the number of permutations was set at 1000. To manage the output size, a cutoff of 150 GO terms, ranked by FDR, was implemented. All GO enrichment results were semantically reduced by using REVIGO, with a dispensability threshold set at <0.4 [26].

### 2.8 Functional re-clustering of DEGs - subcDEGs

From the filtered DEGs obtained from all pairwise Phase comparisons, semantic similarity measures were calculated to determine the gene function-based clusters in each organ [27]. The optimal numbers of clusters were determined by using dynamicTreeCut with deepSplit = 0 [91]. The gene function-based clusters of DEGs are referred to as the subclustered DEGs (subcDEGs). The subcDEGS were then re-submitted for GO enrichment analysis and reduced for term redundancy as described. subcDEGS that did not yield GO enrichment were excluded from downstream analysis (Additional file 6: Table S5).

To evaluate the changes in gene expression pattern through the different STF Phases, we calculated the log_2_FCs (against 0 h, or fed *ad libitum* control) of the genes included in the aforementioned subcDEGs. A two-sided Wilcoxon Rank test was used to determine the statistical significance between means at p≤ 0.05. The mean variances of the log_2_FCs were regressed against the fasting Phases (Spearman's correlation was used).

### 2.9 Gene expression pattern mining

Unique patterns across the STF Phases were determined from log_2_ (x+1) NC of the genes that composed all the subcDEGs of each organ and that yielded GO enrichment. Pattern mining was performed by using topology overlap matrix-based dissimilarity algorithms (WGCNA); next, the optimal cluster numbers were determined by using dynamicTreeCut with deepSplit = 0.

### 2.10 Network enrichment analysis

A list of 349 unique genes was extracted from the 37 GO terms shared among the top four overlapping organs—BRN, LIV, iBAT, and psWAT—to further investigate the enriched biological processes in the *brain-liver-fats* organ network observed in the fasted mice (Additional file 7: Table S6). The Reactome database [28] and ClueGO [29] were used to determine and visualize the enrichment networks and the conserved protein pathways. The following parameters were used to construct the enrichment network—min/max GO level = 3–20, Number of Genes = 3, Min Percentage = 3, Kappa Score Threshold = 0.4, Sharing Group Percentage = 50—and the statistical significance was set at FDR<0.05.

To evaluate the immune components of the organs in the *brain-liver-fats* organ network (BRN, LIV, iBAT, and psWAT), we retrieved a comprehensive list of immune-related genes in mice from innateDB [30] and Mouse Genome Informatics (MGI; http://www.informatics.jax.org/vocab/gene_ontology/GO:0002376) [31]. A list of 3022 genes was used to identify the immune-related genes in this study.

### 2.11 Literature mining - acumenta Literature Lab (LitLab™)

LitLab™ (Acumenta Biotech, USA) allows the identification of biological and biochemical terms significantly associated with the literature from a gene set. The analysis provides additional meaning to experimentally derived gene and protein data [32]. The LitLab™ database contains current gene, biological, and biochemical references in every indexed PubMed abstract and is updated quarterly (currently at 30 million). LitLab™ calculates the frequencies of the input genes for each of 86,000 terms in the Literature Lab™ database (as of January 20, 2020) and compares the values with those of 1000 random gene sets, containing the same number of genes and literature volume profile as the experimental set, to determine statistical significance (cutoff at p value < 0.0228). LitLab™ comprises four main applications: Term Viewer, PLUS, Editor, and Gene Retriever.

### 2.12 PubMed-based literature searches

A literature search of the PubMed database on “fasting” and “immunity” in mice was conducted by using the following search strategy: (“intermittent fasting” [All Fields] OR “Caloric Restriction (CR)” [MeSH Terms] OR “Food Deprivation” [All Fields] OR “food restriction” [All Fields] OR “fasting" [MeSH Terms] OR “fasting” [All Fields]) AND (“gene expression” [All Fields] OR “Gene Expression” [ MeSH Terms] OR “transcriptome” [All Fields] OR “transcriptome” [MeSH Terms]) AND (“mice” [MeSH Terms] OR “mice” [All Fields]) AND (“2010/01/20” [PDAT]: “2020/01/20” [PDAT]) AND (“Immune System Phenomena” [MeSH Terms] OR “Immunity” [MeSH Terms] OR “Immune System” [MeSH Terms] OR immune [All Fields] OR “immune response” [All Fields] OR “inflammation” [MeSH Terms] OR “inflammation” [All Fields] OR “infection” [All Fields]).

To further evaluate our findings with the current literature, we extracted the gene referenced from the relevant resultant articles by using Gene Retriever™ (Acumenta Biotech). Gene Retriever™ is a data mining solution to retrieve all genes associated with a list of PubMed articles. Gene Retriever processes an input list of PubMed IDs and produces an analysis of the genes mentioned in the title, text, and MeSH tags of each record. Results are then statistically ranked and presented in a spreadsheet to enable quick and comprehensive analyses. Hyperlinks are added within the spreadsheet to enable instant review of the genes or PubMed IDs of interest (Additional file 9: Table S8).

### 2.13 Software

All analyses and graphics were performed and generated in R unless otherwise stated. ClueGO was performed in Cytoscape [33]. Adobe Illustrator (Adobe Inc.) was used to prepare the final figures.

## 3 Results

### 3.1 Multiorgan transcriptomic profiling during STF

To investigate the global gene expression dynamics associated with STF, we used mRNA-seq to profile the transcriptome of nine organs obtained from mice fed *ad* libitum (i.e., 0 h time point), or subjected to five STF durations (2, 8, 12, 18, and 22 h of fasting; n = 3 per time point; Fig. 1A). To minimize the impact of circadian influence on gene expression, food was removed from the fasted groups for 2, 8, 12, 18, and 22 h before tissue collection. The nine organs profiled were as follows: OB, brain (BRN, which includes the telencephalon and diencephalon), cerebellum (CBL), brainstem (BST, which comprises the mesencephalon, pons, and myelencephalon), stomach (STM), liver (LIV), interscapular brown adipose tissue (iBAT), perigonadal white adipose tissue (pgWAT), and posterior-subcutaneous white adipose tissue (psWAT). After quality control (see Methods), we retained 157 of 162 samples (97%) for downstream analyses. After applying a cutoff of >10 normalized counts in at least three samples, we found that 13,129–15,012 genes were expressed per organ (Additional file 1: Fig. S1; Additional file 2: Table S1). Next, we performed principal component analysis on the genes expressed across all samples (9420) and found segregation primarily according to organ type and in four clusters across the first three Principal Components: one cluster comprised all the nervous system samples (OB, BRN, CBL, BST), one cluster comprised all the adipose tissues (iBAT, pgWAT, psWAT), and the two remaining clusters comprised STM and LIV, respectively (Figure 1B, Additional file 1: Fig. S2a). Principal component analyses made on all samples from each organ also yielded no clear separation by fasting time (Additional file 1: Fig. S2b). Hierarchical clustering analysis (HCA) of the union of the top 100 most abundant genes expressed across all samples also resulted in similar sample clustering (Fig. 1C). Together, these results indicate that organ clustering is primarily driven by anatomical similarity, and this result is consistent with the findings in the literature [[34], [35], [36]].

### 3.2 Data-driven clustering shows differential temporal effects of STF among organs

To examine the temporal impact of STF in each organ, we employed a data-driven approach to identify gene expression-dependent fasting clusters across all samples. To achieve this objective, we defined the HVGs within each organ. We then calculated, for each organ separately, the optimal number of clusters into which all samples were segregated, based on the expression of organ-specific HVGs (see Methods and Additional file 1: Fig. S1). We observed that for most organs (BRN, CBL, BST, STM, LIV, and iBAT), samples were grouped in three clusters, which we designated Phase I, II, and III (Figure 2A; Additional file 3: Table S2). Samples from OB and psWAT were grouped in two clusters (Phases I and II), which seemingly cycled throughout the 22 h. Notably, the Phases are populated by combinations of samples from multiple fasting times, and samples from the same fasting time are not always integrated into the same fasting Phase, which could occur because of interindividual differences. Moreover, the cyclical transcriptomic pattern observed in OB and psWAT is reminiscent and consistent with other cyclical physiological or metabolic responses occurring in these organs during fasting [[37], [38], [39]]. In contrast with these results, we did not identify robust clusters when using the samples from pgWAT (thus excluding it from our downstream analyses). Although intriguing, that pgWAT and psWAT show differential gene expression dynamics to STF is consistent with other studies, because different adipose depots are functionally distinct and can display differential transcriptomic responses to fasting [40,41]. Additional correlation analysis for all possible pairwise comparisons between the samples within a given organ further supports these results (Additional file 1: Fig. S3). Together, these results show that the temporal effect of STF varies primarily in the function of the organ type, and to a lesser extent in the function of the interindividual variability.

To gain further insights into the transcriptomic changes associated with STF, we next performed differential expression analysis. We used two methods to determine the DEGs: in a pairwise manner between each time point against zero (data not shown), and in a pairwise manner between the aforementioned Phases. A comparative analysis between both sets of DEGs revealed that both methods yield similar numbers of DEGs (Additional file 1: Fig. S4). As in the aforementioned PCA analysis, (Figure 1B and Figs. S2a and b) it was revealed that the samples do not segregate according to fasting time, and we used only the DEGs determined from all possible pairwise comparisons between the fasting Phases identified for each organ in our downstream analyses (Additional file 4: Table S3). We observed that the number of DEGs identified for the different pairwise Phase comparisons varies greatly between organs (Fig. 2B). Among the six organs displaying three Phases, the highest number of DEGs identified in BRN, CBL, and STM were for the Phase I versus III comparison, and for the remaining three organs (BST, LIV and iBAT), the highest numbers were for the Phase II versus III comparison. Similarly, the total number of non-overlapping DEGs identified for each organ also varied greatly (Fig. 2C), with OB displaying the lowest quantity of DEGs (38), and the adipose tissues iBAT and psWAT showed the highest number of DEGs (3822 and 3851, respectively). This analysis yielded multiple sets of known and newly identified genes affected by STF. For instance, between Phases I and III, we found decreasing expression levels of *Nrg4*, *Lep*, and *Ucp1* in psWAT; decreasing expression levels of *Reg1* in STM; and increased expression of *Egr1, Fam107a, Map3k6,* and *Mt1* in the LIV (Figure 2C; Additional file 5: Table S4); and these results are consistent with other STF studies in rodents [11,20,[42], [43], [44], [45]]. In summary, by allowing the samples to cluster in an organ-specific manner based on the HVGs expression, we detected the differential temporal effects of STF on the transcriptional profiles of multiple organs.

### 3.3 Organ-specific modulations by STF

**Figure 3:**
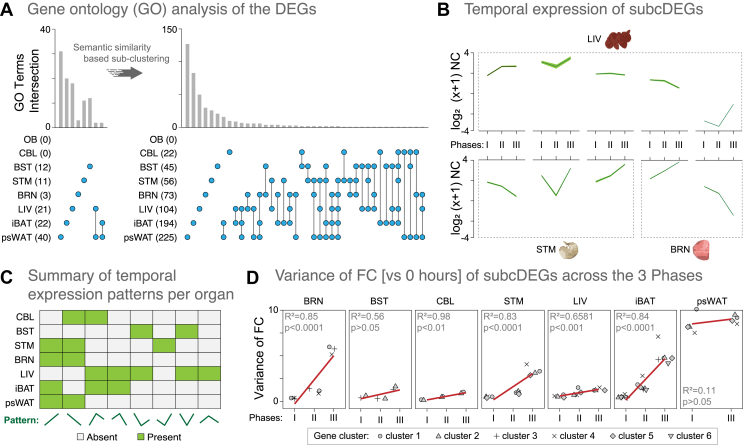
**Functional re-clustering of DEGs increased the identification of the biological perturbations and the unique gene expression patterns in organs of the fasted mice.A)** The number of gene ontology (GO) terms and those shared among organs increased after applying a semantic similarity based sub-clustering algorithm to the filtered DEGs. GO analysis for the original filtered DEGs and for the subcDEGs are represented in the left and right panels, respectively. The number and nature of the GO intersections are denoted by the vertical bar graph and the line-dots, respectively. Number of GOs enriched from the filtered DEGs and subcDEGs are noted in parenthesis in front of the organ acronym. **B)** The unique temporal expression patterns of the genes comprised in the subcDEGs from three select organs (LIV, STM, BRN) across the fasting Phases (from Figure 2A). mRNA expression levels are represented on a log_2_ (x+1) scale of mean normalized counts (NC) (dark green line) ± SD (light green shadow). **C)** Summary of the unique temporal expression patterns of the genes comprised in the subcDEGs in the seven organs. A total of eight different temporal expression patterns are present (green). **D)** Mean variance of the log_2_ fold change (FC) vs. timepoint 0 h of the genes in all the subcDEGs across the three fasting Phases. Linear regression analysis (red line) was performed for each organ, and the R^2^ and p-values are depicted in each panel. Up to six gene clusters, i.e. subcDEGs, have been identified among the seven organs; the cluster number is indicated at the bottom of the panel. Organ acronyms are as in Figure 1.

To obtain insights into the functional roles of the hundreds of DEGs we have identified, we combined multiple strategies to perform GO enrichment analyses (see Methods). To focus our analysis on the genes most impacted by STF, for organs yielding >500 DEGs (i.e., BRN, STM, iBAT, and psWAT), we focused our downstream analyses on the top and bottom 12.5% of the log_2_FC ranked genes. Hereafter, DEGs refer to these filtered DEGs (Additional file 1: Fig. S4b). The biological pathway analysis returned significant results for psWAT, iBAT, LIV, BST, STM, and BRN but not for CBL and OB (Figure 3A). STF appeared to induce changes in unique biological processes among the organs, with only two overlapping GO terms between LIV-psWAT and iBAT-psWAT, respectively (Figure 3A, left panel). Notably, the relatively small number of GO terms shown reflects the stringent thresholds and semantic reduction applied (see Methods); thus, only the most relevant and non-redundant terms were kept.

GO semantic similarity provides the basis for a functional comparison of gene products and is widely used in bioinformatics applications (e.g., cluster analysis of genes) [46,47]. To improve the sensitivity and coverage of our GO enrichment analyses, we subclustered the organ-specific DEGs on the basis of the semantic similarity of their associated GO terms (see Methods); hereafter, referred to as the subclustered DEGs (subcDEGs). We then performed GO enrichment for each of the multiple subcDEGs groups created for each organ; subcDEGs that did not yield enrichment of GO were excluded from downstream analysis. This new approach yielded a 5- to 24-fold increase in the enriched GO terms for all organs except OB, for which we still did not identify enriched pathways, excluding it from further analysis (Figure 3A, right panel; Additional file 6: Table S5). Notably, 22 GO terms were returned for CBL, which previously had no significant enrichments, and for BRN, the annotation coverage increased 24-fold, from 3 to 73 GO terms.

To validate this new approach, we used a semantic network to visualize the GO terms enriched in LIV, which is the most well-studied organ in the context of fasting (Additional file 1: Fig. S5). Among those, we found several GO terms consistent with previous fasting studies, including carboxylic acid biosynthetic process, lipid storage, lipid homeostasis, xenobiotic metabolism, regulation of protein stability, and regulation of protein localization to a membrane [13,16,48]. We also identified other novel pathways enriched by STF, such as wound healing, sensory perception of pain, and positive regulation of vasculature development, highlighting the discovery potential of this approach. In summary, by functionally re-clustering the DEGs, we substantially increased both the organ-specific GO annotation coverage and the probability of discovering novel pathways associated with STF.

We then asked how the genes in each of the subcDEGs that provided GO enrichment varied over STF duration. To achieve this objective, we extracted and summarized the unique expression patterns of the genes from the collective subcDEGs across Phases in each organ (Figure 3B,C). In general, the non-brain organs exhibited a relatively higher number of distinct cluster expression patterns, suggesting comparatively higher dynamics in their gene expression responses to STF. As expected, only two patterns were found in psWAT (explained by two Phases).

To further analyze these gene expression patterns, we calculated the variance of the mean FC versus the timepoint 0 h (i.e., fed *ad libitum*) of the genes in all the subcDEGs across the three Phases and performed regression analysis (Fig. 3D). We found positive correlations of FC variance with STF in most organs, except for BST and psWAT, where the relationships were non-significant. STM, BRN, and iBAT exhibited a noticeably higher degree of FC variances with STF (i.e., the slope of the regression) than those of psWAT, CBL, and LIV did, suggesting greater differential regulation of their transcriptional profiles as STF progressed. Together, these results provide a second line of evidence that different organs show different dynamics in their transcriptional programs to STF. Indeed, intermittent fasting and dietary restriction induce different metabolic trade-offs and organ-specific changes in bioenergetics and the redox state in mice [49,50].

### 3.4 Enrichment network reveals key biological processes conserved among the *brain-liver-fats* organ network in the fasted mice

**Figure 4:**
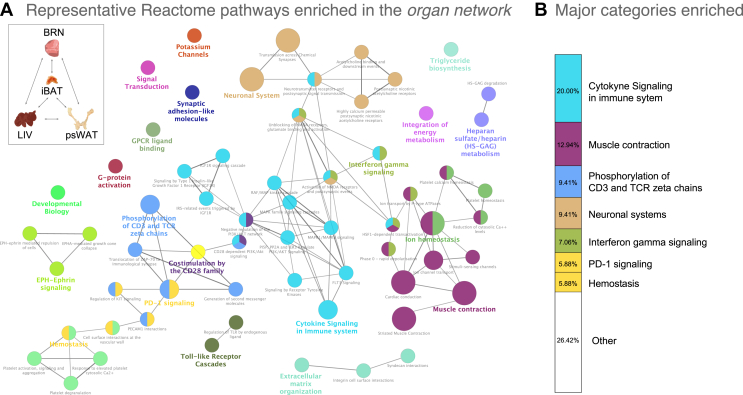
**Enrichment network reveals key biological processes conserved among the *brain-liver-fat* network in the fasted mice**. **A)** Reactome enrichment network of 63 reaction pathways (nodes) and 70 connections (edges), resulting from 188 of 349 genes that were extracted from the 37 overlapping GO terms among BRN, LIV, iBAT and psWAT and passed the significance selection criteria (FDR<0.05). The node color indicates biologically similar reactions, and the size reflects the number of genes contributing to the pathway. If the reaction pathway shares 50% or more of the contributing genes, then they are connected by an edge. The representative nodes (based on FDR) are indicated by the colored texts. On the top left, a schematic representation of the four organs composing the *brain-liver-fats* organ network, and all of their possible interactions (bidirectional arrows). **B)** The representative categories and percentages of the Reactome pathways enriched from the 188 genes obtained from the *brain-liver-fats* organ network. The categories and color are the same as in panel a. Organ acronyms are as in Figure 1.

By functionally re-clustering the DEGs, we also improved the extent of GO terms shared among organs (Fig. 3A). We identified four highly overlapping organs (LIV, BRN, iBAT, and psWAT) based on their enriched biological pathways (Additional file 7: Table S6). Hereafter, we refer to this organ group as the *brain-liver-fats organ network.* To further our understanding of the biological implications of STF among this organ network, we performed network enrichment analysis against the Reactome database with the 349 genes integrating the 37 shared GO terms. The network enrichment of 63 reaction pathways (nodes), with 70 connections (edges), resulting from 188 of the 349 genes that passed the significance selection criteria (see Methods) shows that immune-related pathways are 48% of the total enriched categories, followed by muscle contraction (12.94%) and the neuronal system (9.41%; Figure 4A,B; Additional file 8: Table S7).

To better understand the individual organ responses in the collective network, we represented the gene numbers and proportion of the upregulated genes associated with each of the summarized Reactome terms across the four organs (Additional file 1: Fig. S6a). Additionally, we performed HCA on the gene expression matrix of these 188 genes to highlight the unique and shared STF-induced expression patterns among the four organs (Additional file 1: Fig. S6b). Although we observed organ-specific transcriptional profiles, the temporal effect of STF is consistent within each organ, as demonstrated by the sequential clustering of their respective STF Phases (i.e., Phases I and II cluster closer than Phase III does). Importantly, the differential regulation of the organ DEGs (i.e., percent showing increased expression) indicates that the molecular mechanisms leading to perturbations of the shared biological processes are different.

**Figure 5:**
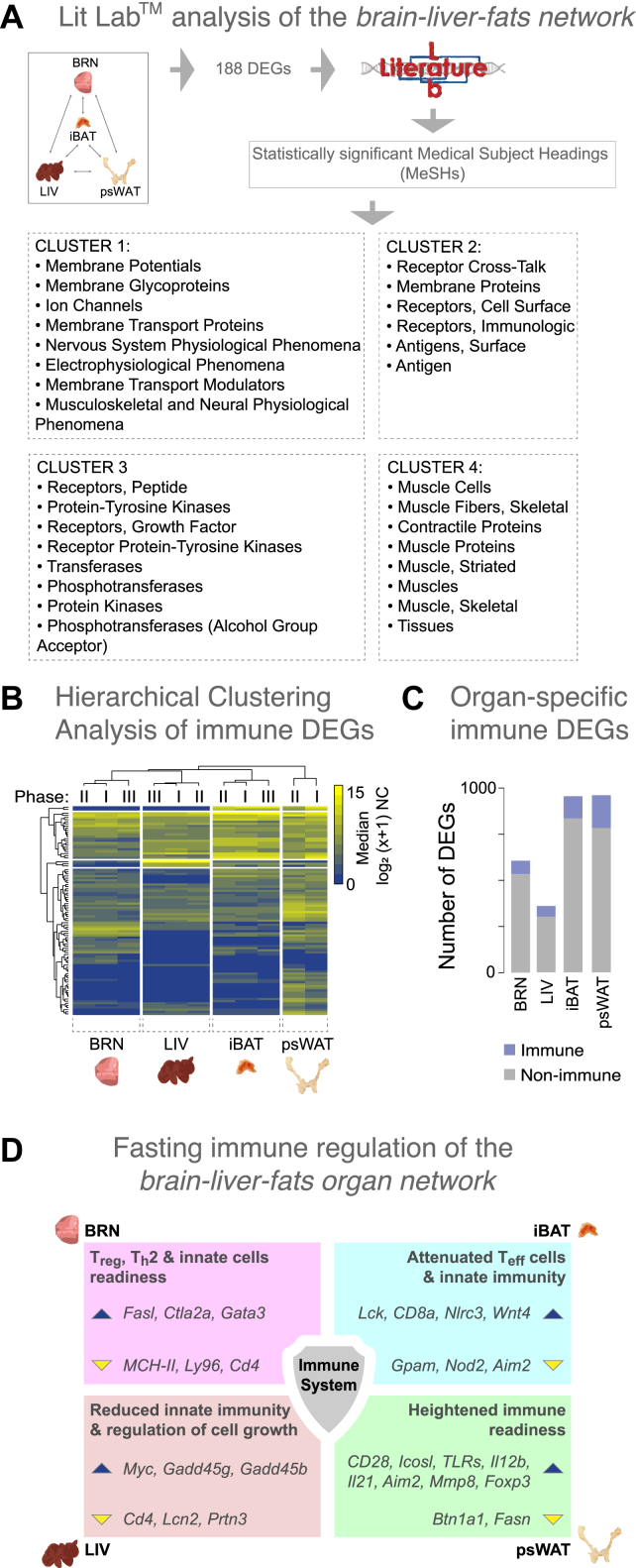
**Current understanding of the biological implications of the *brain-liver-fats* organ network in the fasted mice points to immune related processes.A)** Summary of the significant (cutoff at p-value<0.0228) Medical Subject Heading (MeSH) term clusters associated with the 188 genes among the *brain-liver-fats* organ network were obtained using Literature Lab (LitLab™). **B)** Gene expression hierarchical clustering analysis of the 96 significant genes (FDR<0.05) from an immune-specific GO enrichment analysis (data not shown) of the 349 genes extracted from the 37 overlapping GO terms from BRN, LIV, iBAT and psWAT. Median mRNA expression levels are represented on a log_2_ (x+1) scale of normalized counts (NC) (0 - not expressed; 15 - highly expressed) for the fasting Phases identified in each organ (see Figure 2). **C)** Proportion of immune-specific genes (blue) queried using the innateDB and Mouse Genome Database, among all DEGs of BRN, LIV, iBAT and psWAT. **D)** The immune system in the context of short-term fasting – a summary schematic highlighting the key changes in the immune regulation of the *brain-liver-fats* organ network during fasting. A brief statement, and example of genes, describing the type of immune change are shown for each organ. The blue and yellow triangles indicate increased and decreased gene expression, respectively. Organ acronyms are as in Figure 1.

To assess the known biological effects of STF-induced changes among the *brain-liver-fats* organ network, we used Literature Lab (LitLab™) to perform an association analysis of the 188 aforementioned DEGs mentioned with the scientific literature. In summary, LitLab™ queried the PubMed database (30 million abstracts, January 20 of 2020) for articles associated with the gene list of interest and returned the tagged medical subject headings (MeSHs) with statistical significance. This analysis yielded four MeSH term clusters that mirrored the four major network-derived pathway categories identified in our previous analysis (Figure 5A). We then summarized the significant physiology and pathway-specific MeSH terms returned and identified associations with the *brain-liver-fats* organ network with the immune and nervous systems, pain tolerance, and other pathways and physiological parameters (Additional file 1: Fig. S7a). In summary, the corroborative literature findings support the robustness of our network results, indicating relevant conserved changes in pathway connectivity among the *brain-liver-fats* organ network in response to STF.

### 3.5 STF modulates immune-specific transcriptional programs in the *brain-liver-fats* organ network

We found that among the 349 DEGs extracted from the overlapping GO terms of the *brain-liver-fats* organ network, 42% (119) are annotated as immune-specific (see Methods). To investigate what aspect of the immune system is centrally involved in the organ network, we reconstructed the enrichment network by using an immune-specific GO database (ClueGO). After threshold corrections, 96 genes were retained (Fig. 5B), which resulted in an enrichment of 61 immune-related GO pathways, with 156 connections (data not shown). The general categories and the proportions of the immune enrichment showed that T cell regulation processes account for more than 52% of the overall processes, followed by leukocyte differentiation (12.5%) and microglial cell activation (9.75%; Additional file 1: Fig. S7b). To explore the individual organ responses in the collective network, we represented the gene numbers and the proportion of the upregulated genes associated with each of the summarized immune-specific GO terms across the four organs (Additional file 1: Fig. S7c). These results provide a strong indication that T cell regulation, among other immune processes, is a critical mediator of STF in the *brain-liver-fats* organ network. The differential gene expression changes again highlight that the immune processes are modulated differently among the four organs of this organ network.

To better understand the immune-regulated changes in the individual organs of the *brain-liver-fats* organ network, we asked this question: What is the proportion of immune-related genes among the DEGs for each organ (i.e., the filtered DEGs from the all pairwise Phase comparisons; Additional file 5: Table S4)? Using innateDB and Mouse Genome Database as references, we found that the percentages of immune-specific genes among the DEGs ranged from 11.8% in BRN to 18.8% in psWAT (Fig. 5C). We then identified the top five upregulated and downregulated immune-related genes among the DEGs from the four organs and evaluated their literature-supported functions (Additional file 1: Fig. S8, Fig. S9). We found that nearly one third (11) of those top 40 DEGs were part of the enriched Reactome pathways network (Fig. 4A).

Next, we examined the immune-specific genes among the DEGs to infer the immunological status of the four organs. In the psWAT, higher expression of *CD28*, *Icosl*, *TLRs*, *Il12b*, *Il21, Aim2*, and *Mmp8* (1.5–5.4 Log_2_FC) and lower expression of *Btn1a1* (−4.3 Log_2_FC) in psWAT (Additional file 1: Fig. S10) suggest a proinflammatory activation/environment albeit the absence of infection. Concomitantly, *Foxp3* (1.6 Log_2_FC), which promotes T cell differentiation to Treg, was expressed at higher levels, possibly to prevent overt immune activation. *Fasn* is essential for TLR4 activation in macrophages [51], and its lower expression has been linked to *Nlrp3* inflammasome inhibition and decreased production of the proinflammatory cytokine precursor pro-Il1b [52]. Together with *Foxp3* expression, lower expression of *Fasn* (−2.8 Log_2_FC) suggests a regulated heightened state of immune readiness in psWAT during short-term energy loss. We also observed an increased expression of *Cxcr2*10 (2.36 Log_2_FC), which may be related to reducing adipogenesis [53,54], as a part of the emerging non-conventional functions of chemokine receptors.

In iBAT, the expression of *Lck* (promotes CD8 memory T cells) and CD8a (T cell coreceptor for recognition of antigen) increased by 2.8 and 3.7 log_2_FC, respectively. By contrast, *Gpam*, essential for Cd4 T cell metabolic activation [55], was expressed at 2.6 Log_2_FC lower levels. The components of the Nod-like receptor pathway, *Nod2,* and *Aim2* were also expressed at lower levels (−1.6 and −4.4 Log_2_FC, respectively). *Nlrc3*, an NLR decoy/attenuator shown to attenuate inflammation in myeloid cells, showed increased expression (2.3 log_2_FC). *Wnt4* has been shown to suppress dendritic cell responsiveness [56] and displayed increased expression levels by 3.3 Log_2_FC. Overall, in iBAT, the effector T cell and innate signaling were reduced, suggesting an anti-inflammatory state during STF.

In BRN, we observed higher expression of FAS ligand (1.9 log_2_FC) and lower expression (−4.1 log_2_FC) of *Ly96* (assists immune response via TLR4; Additional file 1: Fig. S10), suggesting immune response priming in the absence of MCHII-antigen peptide presentation. Given the increase in *Gata3* (1.9 log_2_FC), Cd4 activation was deemed reduced, and T2 differentiation was favored. *Ctla2a* expression also increased (3.2 log_2_FC), supporting the bias toward memory and regulated immune response in the brain. Collectively, these immune gene changes suggest that in BRN, STF induced the maintenance of higher innate cell activity, which will probably preserve organ integrity, through the promotion of less destructive effector mechanisms.

In LIV, the expression of *Cd4* and *Lcn2* (mediators of the innate immune response to bacteria) decreased with the fasting time (−1.2 and −3.7 Log_2_FC, respectively, Additional file 1: Fig. S10). By contrast, the expression of *Myc*, which affects cell growth, B cell proliferation, and stem cell renewal, was increased 2.7 Log_2_FC. The *Gadd45* family proteins are upregulated under cellular stress [57] and are involved in the activation of S and G2/M checkpoints [58]. Both the gamma and beta forms are critical in the development of pathogenic effector T cells, and their deficiency in mice leads to lymphoproliferative syndrome and systemic lupus erythematosus [59]. Downregulation of *Gadd45g* contributes to the pathogenesis of hepatocellular carcinoma in both mice and humans [60]. In T cells, *Gadd45b* is a critical mediator for Th1 response to infection [61]. We observed increased expression of 2.07 and 1.54 Log_2_FC for *Gadd45g* and *Gadd45b*, respectively, which is likely an attempt to diminish cellular metabolic activities in response to the stress induced by STF. Fasting-induced increases in *Gadd45b* expression are a liver-specific molecular event promoting adaptive metabolic function in mice [62] and are well in line with the role that *Gadd45g* plays as a cold-induced activator of BAT thermogenesis [63].

Finally, to obtain a second line of evidence that STF affects the transcriptional dynamics of the immune system, we assessed the literature on the collective topics of fasting-related processes, mouse, gene expression, and immune-related processes (see Methods). This search yielded 241 peer-reviewed articles published over ten years. Upon manual inspection, we selected 52 relevant articles for which we then extracted the tagged/associated genes by using Gene Retriever™ (Additional file 9: Table S8). The 151 referenced genes tagged in more than one article included 10% of the genes in the *brain-liver-fats* organ network and 23% of the DEGs for BRN, LIV, iBAT, and psWAT. In other words, a small fraction of the immune transcriptional dynamics we observe during STF in mouse has been reported in a range of other fasting studies, suggesting that some of the molecular processes triggered by fasting might be independent of its duration. This analysis also shows that the vast majority of our findings are novel, highlighting the tremendous discovery potential of the experimental approach we used in this study. In summary, our study expands the knowledge of the molecular processes and pathways shared and modulated in a multiorgan network during short-term energy loss.

## 4 Discussion

Here, we performed mRNA-seq on multiple organs of mice subjected to various periods of STF to understand the molecular mechanisms and biological processes related to short-term energy loss. Our results provide a comprehensive resource on the global mRNA expression changes during STF. We recapitulated some reported physiological/molecular effects from a wide range of longer-term or intermittent fasting studies while providing additional insights into new molecular modulators of fasting in a multiorgan network. Additionally, we present an intuitive analytical method to extrapolate meaningful biological implications from dynamic transcriptomic changes at the organ and system level.

### 4.1 The analytical approach

Gene expression is highly dynamic, in part because of organ-specific expression patterns and biological variation among individuals [64]. The conventional approach to analyzing dynamic transcriptional responses is to infer the enrichment of biological pathways from gene expression changes at multiple time points by analyzing each time point individually. The implicit assumption that the biological processes are independent—the lack of biological dependency across the time points—limits the ability to pinpoint changes at a pathway level in a biological system [65]. To circumvent this limitation, we used HVGs across our dataset as determinants to group samples that best represent their temporal characteristics induced by STF. This data-driven approach allowed us to maximize the detection of the differential temporal gene expression changes informative of, but not constrained by, the experimental time points, which are of particular importance at the multiorgan level. Given the differential prioritization in energetic re-allocation between organs during short-term energy loss [49,50], we logically assume a certain level of asynchrony in their temporal gene expression. Using this approach, we characterized the differential temporal effects of STF on the global gene expression patterns among the nine mouse tissues.

Although we observed FC direction and magnitude in some genes that have been reported in the literature, we did not observe some known changes, such as a significant increase of *Fgf21* transcript in LIV with increased fasting duration. FGF21 has been shown to act as a negative feedback signal to terminate growth hormone (GH)-stimulated regulation of glucose and lipid metabolism under fasting conditions [66]. In mice, high levels of FGF21 suppress the activity of GH and reduce the production of insulin-like growth factors (IGF) [67]. In line with the literature, we observed drastic reductions in *Gh* (−4.68 log_2_FC) and *Igf2* (−3.06 log_2_FC) expressions in BRN and a trend of increasing raw expression of *Fgf21* in LIV with STF (Additional file 2: Table S1). Recently, FGF21 was demonstrated to be partially required for appropriate gene expression during the fed to fasted transition in mice [68]. FGF21-KO mice and pharmacological blockage of the FGF21 axis did not profoundly disrupt the physiological response to fasting. Additionally, STF (<60 h) did not affect plasma FGF21 level in lean human subjects; however, the mRNA expression of FGF21 receptors (KLB) was decreased in the subcutaneous WAT from both lean and obese subjects [69]. In concordance, we observed a −3.30 log_2_FC decrease in *Klb* expression in psWAT by STF. Thus, both study heterogeneity and biology may have contributed to these observed gene expression differences in the context of fasting.

An analysis of multiple CR studies in mice detected relatively few genes that exhibited a consistent expression response across numerous experimental conditions [10]. Thus, relying on specific subsets of DEGs is unlikely to be a method to find common biological processes and to provide a meaningful representation of systemic effects. By contrast, a high-level approach, for example, GO enrichment analysis, is more likely to reveal common biological pathways [70]. Gene set analyses are now standard practice for the functional annotation of gene lists. However, the enrichment bias for multifunctional genes (i.e., frequently represented in GO terms) is an inherent challenge [71] and drives the generation of biologically non-specific and highly fragile significances in genomic studies [72]. Additionally, the amount of redundancy and overlaps in GO terms can make result summarization challenging.

To address these issues, we clustered the experimentally derived gene list from each organ by using the semantic similarity of their functional annotations (i.e., subcDEGs). We then reduced the redundancy of the resulting GO terms by using semantic uniqueness and retained only the most relevant and profoundly affected biological pathways (see Methods). Despite the stringency of our enrichment methods, we obtained increased and sufficient organ overlaps that allowed for the investigation of the biologically relevant events occurring at the multiorgan level.

### 4.2 A *brain-liver-fats* organ network modulated by STF

We hypothesized that the systemic effects of STF would be, at some level, exerted through biological perturbations shared among multiple organs. We identified four organs that highly overlapped in their enriched biological processes, which we called the *brain-liver-fats* organ network. Evidence for crosstalk between different pairs of these four organs has been shown in the context of fasting. For example, during prolonged fasting, PPARα and FGF21 signaling between the brain and liver mediates glucose homeostasis [73]. In mice, fasting-induced glycogen shortage activates a liver-brain-adipose neurocircuitry to facilitate fat utilization [74], and the regulation of food intake and glucose homeostasis by liver glycogen is dependent on the hepatic branch of the vagus nerve [75]. In addition, leptin-mediated interactions between the brain and adipose depots related to the maintenance of systemic energy balance were recently reviewed [76]. These other studies have provided additional lines of evidence for the existence of our proposed *brain-liver-fats* organ network in the context of STF.

Within the organ network, we identified immune-related pathways, muscle contraction, neuronal systems, and signal transductions as the top conserved pathways affected by STF. Additionally, with LitLab™, we found strong associations between the genes that comprised our organ network and pain signaling and physiological response to pain. In line with this, fasting and calorie restriction have an analgesic effect in murine models [[77], [78], [79]], and intermittent fasting was proposed as a non-invasive, inexpensive, and implementable strategy to chronic pain treatment (reviewed in [80]). Key underlying mechanisms in fasting-enhanced neuroplasticity include a short-term corticosterone increase, a reduction in GABAergic inhibition, and an increase in protein chaperons and neurotrophic growth factors such as brain-derived neurotrophic factor (BDNF), which exerts positive effects on neuronal survival and synaptogenesis [[81], [82], [83]]. The BDNF pathway showed a strong association with our organ network gene list, driven by the presence of both *Bdnf* and its receptor, *Ntrk2b* (Additional file 7: Table S6).

As our gene-MeSHs association queries (over 30 million abstracts) went beyond single-study comparisons, the results provided unbiased and statistically significant support for our experimental observations. We recapitulated several known genes reported in literature associated with fasting, gene expression, immunity, and mouse. As a result of the synthesis of the multiorgan transcriptome, we observed that 90% of the genes encompassing the organ network might represent potential novel molecular modulators of the dynamic biological and immunological perturbations in mice subjected to STF.

### 4.3 Immune system during homeostatic perturbations

The physiological response to STF is a consortium of organ adaptation, aimed to preserve the most critical functions amidst a systemic decrease in energy availability. The topic of the immune system acting as a regulator of organismal homeostasis in the absence of infection has been recently reviewed [[84], [85], [86]]. Non-infectious signals, such as physiological perturbation (e.g., cold exposure) and diet metabolites, can regulate the equilibrium between types of immune responses (e.g., intracellular, parasitic, extracellular) [87,88]. These non-canonical modulations of cytokines in the innate and adaptive immune systems have an essential role in regulating complex organ physiology (reviewed in [85]). These studies suggest that immune cells are well-positioned and equipped to sense homeostatic perturbations and relay signals at the systems level in the absence of infection.

In this context, many studies have focused on the neuronal regulation of inflammation, neuroimmune circuits in interorgan communication, and the role immune cells play in the systemic regulation of metabolism and obesity (recently reviewed in [89,90]). For example, macrophage polarization toward a classically (M1-like) activated state is a characteristic of obese adipose tissue [91], and adipose tissue macrophages have remained the primary immune participant studied in the context of obesity since their discovery [92,93]. Non-canonical pathways of macrophage activation through metabolites (i.e., glucose, insulin, and palmitate) also result in a continuum of proinflammatory phenotypes [90]. Moreover, sympathetic neuron-associated macrophages were recently identified and shown to affect norepinephrine (NE)-mediate regulation of thermogenesis of adipose tissue by facilitating NE clearance and shifting to a more proinflammatory state [94]. Furthermore, liver macrophages contribute to insulin resistance independently of their inflammatory status, through the secretion of IGFBP7, a non-inflammatory factor with a high capacity to bind the insulin receptor and induce lipogenesis and gluconeogenesis through the activation of ERK signaling [95].

### 4.4 Immune system in the context of STF

The *brain-liver-fats* organ network described in this study highlights the importance of the immune processes modulated by STF. However, the mechanisms underlying fasting-induced effects on the immune system remain largely unknown, and researchers have only recently started to elucidate this topic [96]. Three studies have demonstrated that monocytes, naïve B cells, and memory CD8 T cells use bone marrow as a refuge during periods of energy reduction to maintain systemic immune-responsiveness [[4], [5], [6]]. These studies also provided new insights into the integrated immunometabolic response in a state of energy deprivation (Fig. 5D).

Among the organ network, we found increased expression of genes that negatively regulate monocyte and macrophage activation (*Tiff2* and *Myc*) and promote regulatory T cell differentiation (*Ctla2a*), B cell differentiation in the bone marrow (*Fzd9*), cytotoxic T cell differentiation (*Cd8a*), suppression of type 2 immunity and inflammation (*Wnt4*), and modulation of neuroinflammation and priming of innate immunity (*S100a8* and *S100a9*; Fig.5D). Moreover, we found that the immune genes showing lower expression values with higher durations of fasting time are involved in the promotion of inflammation (*Ly96*), activation of the innate immune response (*lfi203* and *Lcn2*), negative regulation of T cell proliferation (*Btn1a1*), inhibition of innate immune response to virus infection (*Trim29*), and mediation of inflammasome activation (*Aim2*). Intuitively, the direction or amplitude of the immunological responses to STF in different organs is unlikely to be the same. Notably, we found enriched expression for genes contributing to T cells and the innate response in psWAT, but lower expression levels for inflammation-related genes in iBAT.

Overall, we observed significant increases in the expression of genes involved in T and B cell differentiation and proliferation, suggesting these immune cells are differentiated within the organ network or in circulation. Decreased expression levels of inflammatory markers support a systemic effort to reduce innate immune signals to the adaptive, possibly by blocking cytokine signals and antigen presentation. Intermittent fasting alters T cells’ differentiation bias in the gut, reducing IL-17 producing T cells, and increasing regulatory T cells [97]. Fasting-mimicking diets lessen the severity and symptoms in a multiple sclerosis mouse model and are associated with increased regulatory T cells and reduced levels of proinflammatory cytokines, Th1 and Th17 cells, and antigen-presenting cells [98]. Recently, CR was shown to reverse aging-related proinflammatory effects in old rats [99]. Several proinflammatory markers, such as *Cxcl2*, *S100a8,* and *S100a9*, were downregulated in 30 different cell types by CR in iBAT and psWAT of aging-mice compared with controls, and these results are consistent with the observed regulatory features (i.e., attenuated T_eff_ cell and innate immunity) of iBAT in our study. In psWAT, we observed an increased abundance of the markers with STF; however, increased *Foxp3* and decreased *Fasn* and *Btn1a1* expression levels indicate a coordinated regulation/control of the immune response in the organ. Differences in the sample type studied (single cell vs. bulk tissue) and dietary interventions can explain part of the divergences at the gene level. Thus, investigation of cellular abundance in organs with different STF durations is warranted in further research.

Neutrophils *Lcn2* expression in rats undergoing CR was upregulated compared with rats fed *ad libitum* and was suggested to correspond to the translocation of neutrophils to the bone marrow [99]. In our study, *Lcn2* was markedly reduced by STF in LIV and was interpreted in relation to other studies performed in mice. In mice, *Lcn2* is expressed in multiple tissues, including liver and immune cells (reviewed in [100]), and its decrease may indicate a reduction in neutrophils recruitment [101]. However, the decrease in *Lcn2* can also suggest increased immune sensitivity (i.e., perhaps a step toward a “priming state”), because *Lcn2*−/− mice secreted significantly more proinflammatory mediators in response to LPS compared with the wild type [102]. In longer-term fasting experiments (24 or 48 h), *Lcn2* expression in mice WAT and BAT was upregulated compared with that of the *ad libitum* fed mice [103]. An explanation might be that the state of metabolism is more similar between a longer period of fasting and CR, compared with STF.

In line with the literature, the observed changes in our multiorgan immune-transcriptome are such that the effects of STF reprogram the immune system, allowing a spectrum of cellular differentiation to occur but restricting immediate reactivity. In summary, our study provides evidence of a consortium of organ adaptation to short-term energy deprivation, in which the immune system plays a central role. Furthermore, we added insights into the molecular events of fasting-induced priming of T cell-mediated immunity, underlining a putative multiorgan effort to support the recently reported egress of T cells and B cells to the bone marrow during periods of systemic energy reduction [5,6].

## 5 Conclusions

Although the purpose of our study was not to decipher the molecular communication between organs or investigate the migratory behaviors or composition of immune cells under fasting conditions [[4], [5], [6]], our study highlights the centrality of immune-transcriptomic modulations during STF. Using a combinatorial data analysis approach, we provide evidence for the existence of an organ network, formed by the similarities of their biological processes, and the prominent role of the immune system in sensing and modulating systemic homeostatic perturbations in the absence of infection. Additionally, we provide a valuable transcriptome resource to further expand on the knowledge of the molecular events occurring across multiple organs during short-term energy loss.

## Ethics statement

The use and care of animals used in this study were in accordance with UK Home Office regulations and under a project license approved by the Wellcome Sanger Institute Animal Welfare and Ethical Review Body.

## Data availability

The raw RNA-seq data (Fastq and raw counts files) from the 162 samples were deposited into GEO, under the study accession number GSE149468.

## Funding

This work was supported by Sidra Medicine and Qatar National Research Fund (a member of 10.13039/100007458Qatar Foundation) Program grant JSREP07-016-1-006 (awarded to LRS). The findings herein reflect the work and are solely the responsibility of the authors.

## Author's contributions

LRS, MG, and SSY designed and led the study. LRS, EHA, LSM, KW, MCL performed experiments. SSYH, MM, MLRTS, AS, MG, and LRS analyzed data and SSYH, MG, and LRS interpreted data. DWL, and DC contributed analysis tools and interpreted data. SSYH, MG, and LRS wrote and MM, AS, DC, and DWL commented on the manuscript. All authors have read and approved the final manuscript.

## References

[1] Longo V.D., Panda S. (2016). Fasting, circadian rhythms, and time-restricted feeding in healthy lifespan. Cell Metabolism.

[2] Mattson M.P., Longo V.D., Harvie M. (2017). Impact of intermittent fasting on health and disease processes. Ageing Research Reviews.

[3] Stockman M.-C., Thomas D., Burke J., Apovian C.M. (2018). Intermittent fasting: is the wait worth the weight?. Current Obesity Reports.

[4] Nagai M., Noguchi R., Takahashi D., Morikawa T., Koshida K., Komiyama S. (2019). Fasting-Refeeding impacts immune cell dynamics and mucosal immune responses. Cell.

[5] Collins N., Han S.-J., Enamorado M., Link V.M., Huang B., Moseman E.A. (2019). The bone marrow protects and optimizes immunological memory during dietary restriction. Cell.

[6] Jordan S., Tung N., Casanova-Acebes M., Chang C., Cantoni C., Zhang D. (2019). Dietary intake regulates the circulating inflammatory monocyte pool. Cell.

[7] Intermountain Health Care, Inc. (2017). The fasting II study. https://clinicaltrials.gov/ct2/show/NCT01792986.

[8] Kessler C.S., Stange R., Schlenkermann M., Jeitler M., Michalsen A., Selle A. (2018). A nonrandomized controlled clinical pilot trial on 8 wk of intermittent fasting (24 h/wk). Nutrition.

[9] Stekovic S., Hofer S.J., Tripolt N., Aon M.A., Royer P., Pein L. (2019). Alternate day fasting improves physiological and molecular markers of aging in healthy, non-obese humans. Cell Metabolism.

[10] Swindell W.R. (2009). Genes and gene expression modules associated with caloric restriction and aging in the laboratory mouse. BMC Genomics.

[11] Kim K.-H., Kim Y.H., Son J.E., Lee J.H., Kim S., Choe M.S. (2017). Intermittent fasting promotes adipose thermogenesis and metabolic homeostasis via VEGF-mediated alternative activation of macrophage. Cell Research.

[12] Goldstein I., Baek S., Presman D.M., Paakinaho V., Swinstead E.E., Hager G.L. (2017). Transcription factor assisted loading and enhancer dynamics dictate the hepatic fasting response. Genome Research.

[13] Kinouchi K., Magnan C., Ceglia N., Liu Y., Cervantes M., Pastore N. (2018). Fasting imparts a switch to alternative daily pathways in liver and muscle. Cell Reports.

[14] Rennert C., Vlaic S., Marbach-Breitrück E., Thiel C., Sales S., Shevchenko A. (2018). The diurnal timing of starvation differently impacts murine hepatic gene expression and lipid metabolism – a systems biology analysis using self-organizing maps. Frontiers in Physiology.

[15] Derous D., Mitchell S.E., Green C.L., Wang Y., Han J.D.J., Chen L. (2018). The effects of graded levels of calorie restriction: X. Transcriptomic responses of epididymal adipose tissue. The Journals of Gerontology. Series A, Biological Sciences and Medical Sciences.

[16] Ng G.Y.-Q., Kang S.-W., Kim J., Alli-Shaik A., Baik S.-H., Jo D.-G. (2019). Genome-wide transcriptome analysis reveals intermittent fasting-induced metabolic rewiring in the liver. Dose-Response.

[17] Smith R.L., Soeters M.R., Wüst R.C.I., Houtkooper R.H. (2018). Metabolic flexibility as an adaptation to energy resources and requirements in health and disease. Endocrine Reviews.

[18] Jensen T.L., Kiersgaard M.K., Sørensen D.B., Mikkelsen L.F. (2013). Fasting of mice: a review. Laboratory Animals.

[19] Hakvoort T.B.M., Moerland P.D., Frijters R., Sokolović A., Labruyère W.T., Vermeulen J.L.M. (2011). Interorgan coordination of the murine adaptive response to fasting. Journal of Biological Chemistry.

[20] Schupp M., Chen F., Briggs E.R., Rao S., Pelzmann H.J., Pessentheiner A.R. (2013). Metabolite and transcriptome analysis during fasting suggest a role for the p53-Ddit4 axis in major metabolic tissues. BMC Genomics.

[21] Kim D., Pertea G., Trapnell C., Pimentel H., Kelley R., Salzberg S.L. (2013). TopHat2: accurate alignment of transcriptomes in the presence of insertions, deletions and gene fusions. Genome Biology.

[22] Wang L., Wang S., Li W. (2012). RSeQC: quality control of RNA-seq experiments. Bioinformatics.

[23] Carlson M. (2019). org.Mm.eg.db. Genome wide annotation for Mouse.

[24] Yu G., Wang L.-G., Han Y., He Q.-Y. (2012). clusterProfiler: an R Package for comparing biological themes among gene clusters. OMICS: A Journal of Integrative Biology.

[25] Huang D.W., Sherman B.T., Lempicki R.A. (2009). Bioinformatics enrichment tools: paths toward the comprehensive functional analysis of large gene lists. Nucleic Acids Research.

[26] Supek F., Bošnjak M., Škunca N., Šmuc T. (2011). REVIGO summarizes and visualizes long lists of gene ontology terms. PloS One.

[27] Yu G., Li F., Qin Y., Bo X., Wu Y., Wang S. (2010). GOSemSim: an R package for measuring semantic similarity among GO terms and gene products. Bioinformatics.

[28] Croft D., Mundo A.F., Haw R., Milacic M., Weiser J., Wu G. (2014). The Reactome pathway knowledgebase. Nucleic Acids Research.

[29] Bindea G., Mlecnik B., Hackl H., Charoentong P., Tosolini M., Kirilovsky A. (2009). ClueGO: a Cytoscape plug-in to decipher functionally grouped gene ontology and pathway annotation networks. Bioinformatics (Oxford, England).

[30] Breuer K., Foroushani A.K., Laird M.R., Chen C., Sribnaia A., Lo R. (2013). InnateDB: systems biology of innate immunity and beyond—recent updates and continuing curation. Nucleic Acids Research.

[31] Bult C.J., Blake J.A., Smith C.L., Kadin J.A., Richardson J.E. (2019). Mouse genome database group, 2019. mouse genome database (MGD). Nucleic Acids Research.

[32] Febbo P.G., Mulligan M.G., Slonina D.A., Stegmeir K., Di Vizio D., Martinez P.R. (2007). Literature Lab: a method of automated literature interrogation to infer biology from microarray analysis. BMC Genomics.

[33] Shannon P., Markiel A., Ozier O., Baliga N.S., Wang J.T., Ramage D. (2003). Cytoscape: a software environment for integrated models of biomolecular interaction networks. Genome Research.

[34] Pan Q., Shai O., Misquitta C., Zhang W., Saltzman A.L., Mohammad N. (2004). Revealing global regulatory features of mammalian alternative splicing using a quantitative microarray platform. Molecular Cell.

[35] Su A.I., Wiltshire T., Batalov S., Lapp H., Ching K.A., Block D. (2004). A gene atlas of the mouse and human protein-encoding transcriptomes. Proceedings of the National Academy of Sciences.

[36] Zhang W., Morris Q.D., Chang R., Shai O., Bakowski M.A., Mitsakakis N. (2004). The functional landscape of mouse gene expression. Journal of Biology.

[37] Caba M., Pabello M., Moreno M.L., Meza E. (2014). Main and accessory olfactory bulbs and their projections in the brain anticipate feeding in food-entrained rats. Chronobiology International.

[38] Mercer S.W., Williamson D.H. (1988). The influence of starvation and natural refeeding on the rate of triacylglycerol/fatty acid substrate cycling in brown adipose tissue and different white adipose sites of the rat in vivo. The role of insulin and the sympathetic nervous system. Bioscience Reports.

[39] Nolasco N., Juárez C., Morgado E., Meza E., Caba M. (2012). A circadian clock in the olfactory bulb anticipates feeding during food anticipatory activity. PloS One.

[40] Palou M., Sánchez J., Priego T., Rodríguez A.M., Picó C., Palou A. (2010). Regional differences in the expression of genes involved in lipid metabolism in adipose tissue in response to short- and medium-term fasting and refeeding. The Journal of Nutritional Biochemistry.

[41] Schoettl T., Fischer I.P., Ussar S. (2018). Heterogeneity of adipose tissue in development and metabolic function. Journal of Experimental Biology.

[42] Lambrecht N.W.G., Yakubov I., Sachs G. (2007). Fasting-induced changes in ECL cell gene expression. Physiological Genomics.

[43] Sokolović M., Sokolović A., Wehkamp D., van Themaat E.V.L., de Waart D.R., Gilhuijs-Pederson L.A. (2008). The transcriptomic signature of fasting murine liver. BMC Genomics.

[44] Tang H.-N., Tang C.-Y., Man X.-F., Tan S.-W., Guo Y., Tang J. (2017). Plasticity of adipose tissue in response to fasting and refeeding in male mice. Nutrition & Metabolism.

[45] Wu V., Sumii K., Tari A., Sumii M., Walsh J.H. (1991). Regulation of rat antral gastrin and somatostatin gene expression during starvation and after refeeding. Gastroenterology.

[46] Bolshakova N., Azuaje F., Cunningham P. (2005). A knowledge-driven approach to cluster validity assessment. Bioinformatics.

[47] Wolting C., McGlade C.J., Tritchler D. (2006). Cluster analysis of protein array results via similarity of Gene Ontology annotation. BMC Bioinformatics.

[48] Chi Y., Youn D.Y., Xiaoli A.M., Liu L., Pessin J.B., Yang F. (2020). Regulation of gene expression during the fasting–feeding cycle of the liver displays mouse strain specificity. Journal of Biological Chemistry.

[49] Książek A., Konarzewski M. (2012). Effect of dietary restriction on immune response of laboratory mice divergently selected for basal metabolic rate. Physiological and Biochemical Zoology: PBZ.

[50] Chausse B., Vieira-Lara M.A., Sanchez A.B., Medeiros M.H.G., Kowaltowski A.J. (2015). Intermittent fasting results in tissue-specific changes in bioenergetics and redox state. PloS One.

[51] Carroll R.G., Zasłona Z., Galván-Peña S., Koppe E.L., Sévin D.C., Angiari S. (2018). An unexpected link between fatty acid synthase and cholesterol synthesis in proinflammatory macrophage activation. Journal of Biological Chemistry.

[52] Moon J.-S., Lee S., Park M.-A., Siempos I.I., Haslip M., Lee P.J. (2015). UCP2-induced fatty acid synthase promotes NLRP3 inflammasome activation during sepsis. Journal of Clinical Investigation.

[53] Dyer D.P., Nebot J.B., Kelly C.J., Medina-Ruiz L., Schuette F., Graham G.J. (2019). The chemokine receptor CXCR2 contributes to murine adipocyte development. Journal of Leukocyte Biology.

[54] Kusuyama J., Komorizono A., Bandow K., Ohnishi T., Matsuguchi T. (2016). CXCL3 positively regulates adipogenic differentiation. Journal of Lipid Research.

[55] Faris R., Fan Y.-Y., De Angulo A., Chapkin R.S., deGraffenried L.A., Jolly C.A. (2014). Mitochondrial glycerol-3-phosphate acyltransferase-1 is essential for murine CD4(+) T cell metabolic activation. Biochimica Et Biophysica Acta.

[56] Hung L.-Y., Johnson J.L., Ji Y., Christian D.A., Herbine K.R., Pastore C.F. (2019). Cell-intrinsic Wnt4 influences conventional dendritic cell fate determination to suppress type 2 immunity. Journal of Immunology (Baltimore, Md: 1950).

[57] Takekawa M., Saito H. (1998). A family of stress-inducible GADD45-like proteins mediate activation of the stress-responsive MTK1/MEKK4 MAPKKK. Cell.

[58] Vairapandi M., Balliet A.G., Hoffman B., Liebermann D.A. (2002). GADD45b and GADD45g are cdc2/cyclinB1 kinase inhibitors with a role in S and G2/M cell cycle checkpoints induced by genotoxic stress. Journal of Cellular Physiology.

[59] Liu L., Tran E., Zhao Y., Huang Y., Flavell R., Lu B. (2005). Gadd45 beta and Gadd45 gamma are critical for regulating autoimmunity. Journal of Experimental Medicine.

[60] Zhang L., Yang Z., Ma A., Qu Y., Xia S., Xu D. (2014). Growth arrest and DNA damage 45G down-regulation contributes to Janus kinase/signal transducer and activator of transcription 3 activation and cellular senescence evasion in hepatocellular carcinoma. Hepatology (Baltimore, Md.).

[61] Lu B., Ferrandino A.F., Flavell R.A. (2004). Gadd45beta is important for perpetuating cognate and inflammatory signals in T cells. Nature Immunology.

[62] Fuhrmeister J., Zota A., Sijmonsma T.P., Seibert O., Cıngır Ş., Schmidt K. (2016). Fasting-induced liver GADD45β restrains hepatic fatty acid uptake and improves metabolic health. EMBO Molecular Medicine.

[63] Gantner M.L., Hazen B.C., Conkright J., Kralli A. (2014). GADD45γ regulates the thermogenic capacity of brown adipose tissue. Proceedings of the National Academy of Sciences of the United States of America.

[64] Tan M.H., Li Q., Shanmugam R., Piskol R., Kohler J., Young A.N. (2017). Dynamic landscape and regulation of RNA editing in mammals. Nature.

[65] Khatri P., Sirota M., Butte A.J. (2012). Ten years of pathway analysis: current approaches and outstanding challenges. PLoS Computational Biology.

[66] Chen W., Hoo R.L., Konishi M., Itoh N., Lee P.-C., Ye H. (2011). Growth hormone induces hepatic production of fibroblast growth factor 21 through a mechanism dependent on lipolysis in adipocytes. Journal of Biological Chemistry.

[67] Inagaki T., Lin V.Y., Goetz R., Mohammadi M., Mangelsdorf D.J., Kliewer S.A. (2008). Inhibition of growth hormone signaling by the fasting-induced hormone FGF21. Cell Metabolism.

[68] Antonellis P.J., Hayes M.P., Adams A.C. (2016). Fibroblast growth factor 21-null mice do not exhibit an impaired response to fasting. Frontiers in Endocrinology.

[69] Nygaard E.B., Ørskov C., Almdal T., Vestergaard H., Andersen B. (2018). Fasting decreases plasma FGF21 in obese subjects and the expression of FGF21 receptors in adipose tissue in both lean and obese subjects. Journal of Endocrinology.

[70] Subramanian A., Tamayo P., Mootha V.K., Mukherjee S., Ebert B.L., Gillette M.A. (2005). Gene set enrichment analysis: a knowledge-based approach for interpreting genome-wide expression profiles. Proceedings of the National Academy of Sciences of the United States of America.

[71] Gillis J., Pavlidis P. (2011). The impact of multifunctional genes on “guilt by association” analysis. PloS One.

[72] Ballouz S., Pavlidis P., Gillis J. (2017). Using predictive specificity to determine when gene set analysis is biologically meaningful. Nucleic Acids Research.

[73] Liang Q., Zhong L., Zhang J., Wang Y., Bornstein S.R., Triggle C.R. (2014). FGF21 maintains glucose homeostasis by mediating the cross talk between liver and brain during prolonged fasting. Diabetes.

[74] Izumida Y., Yahagi N., Takeuchi Y., Nishi M., Shikama A., Takarada A. (2013). Glycogen shortage during fasting triggers liver–brain–adipose neurocircuitry to facilitate fat utilization. Nature Communications.

[75] López-Soldado I., Fuentes-Romero R., Duran J., Guinovart J.J. (2017). Effects of hepatic glycogen on food intake and glucose homeostasis are mediated by the vagus nerve in mice. Diabetologia.

[76] Caron A., Lee S., Elmquist J.K., Gautron L. (2018). Leptin and brain–adipose crosstalks. Nature Reviews Neuroscience.

[77] Hargraves W.A., Hentall I.D. (2005). Analgesic effects of dietary caloric restriction in adult mice. Pain.

[78] Liu Y., Ni Y., Zhang W., Sun Y.-E., Ma Z., Gu X. (2017). Antinociceptive effects of caloric restriction on post-incisional pain in nonobese rats. Scientific Reports.

[79] Lee J.-Y., Lee G.J., Lee P.R., Won C.H., Kim D., Kang Y. (2019). The analgesic effect of refeeding on acute and chronic inflammatory pain. Scientific Reports.

[80] Sibille K.T., Bartsch F., Reddy D., Fillingim R.B., Keil A. (2016). Increasing neuroplasticity to bolster chronic pain treatment: a role for intermittent fasting and glucose administration?. The Journal of Pain: Official Journal of the American Pain Society.

[81] Spolidoro M., Baroncelli L., Putignano E., Maya-Vetencourt J.F., Viegi A., Maffei L. (2011). Food restriction enhances visual cortex plasticity in adulthood. Nature Communications.

[82] Mattson M.P., Duan W., Guo Z. (2003). Meal size and frequency affect neuronal plasticity and vulnerability to disease: cellular and molecular mechanisms. Journal of Neurochemistry.

[83] van Praag H., Fleshner M., Schwartz M.W., Mattson M.P. (2014). Exercise, energy intake, glucose homeostasis, and the brain. Journal of Neuroscience: The Official Journal of the Society for Neuroscience.

[84] Caputa G., Castoldi A., Pearce E.J. (2019). Metabolic adaptations of tissue-resident immune cells. Nature Immunology.

[85] Rankin L.C., Artis D. (2018). Beyond host defense: emerging functions of the immune system in regulating complex tissue physiology. Cell.

[86] Veiga-Fernandes H., Freitas A.A. (2017). The S(c)ensory immune system theory. Trends in Immunology.

[87] Shibata N., Kunisawa J., Kiyono H. (2017). Dietary and microbial metabolites in the regulation of host immunity. Frontiers in Microbiology.

[88] Macpherson A.J., de Agüero M.G., Ganal-Vonarburg S.C. (2017). How nutrition and the maternal microbiota shape the neonatal immune system. Nature Reviews Immunology.

[89] Huh J.R., Veiga-Fernandes H. (2019). Neuroimmune circuits in inter-organ communication. Nature Reviews Immunology.

[90] Larabee C.M., Neely O.C., Domingos A.I. (2020). Obesity: a neuroimmunometabolic perspective. Nature Reviews Endocrinology.

[91] Lumeng C.N., Bodzin J.L., Saltiel A.R. (2007). Obesity induces a phenotypic switch in adipose tissue macrophage polarization. Journal of Clinical Investigation.

[92] Weisberg S.P., McCann D., Desai M., Rosenbaum M., Leibel R.L., Ferrante A.W. (2003). Obesity is associated with macrophage accumulation in adipose tissue. Journal of Clinical Investigation.

[93] Xu H., Barnes G.T., Yang Q., Tan G., Yang D., Chou C.J. (2003). Chronic inflammation in fat plays a crucial role in the development of obesity-related insulin resistance. Journal of Clinical Investigation.

[94] Pirzgalska R.M., Seixas E., Seidman J.S., Link V.M., Sánchez N.M., Mahú I. (2017). Sympathetic neuron–associated macrophages contribute to obesity by importing and metabolizing norepinephrine. Nature Medicine.

[95] Morgantini C., Jager J., Li X., Levi L., Azzimato V., Sulen A. (2019). Liver macrophages regulate systemic metabolism through non-inflammatory factors. Nature Metabolism.

[96] Buono R., Longo V.D. (2019). When fasting gets tough, the tough immune cells get going—or die. Cell.

[97] Cignarella F., Cantoni C., Ghezzi L., Salter A., Dorsett Y., Chen L. (2018). Intermittent fasting confers protection in CNS autoimmunity by altering the gut microbiota. Cell Metabolism.

[98] Choi I.Y., Piccio L., Childress P., Bollman B., Ghosh A., Brandhorst S. (2016). A diet mimicking fasting promotes regeneration and reduces autoimmunity and multiple sclerosis symptoms. Cell Reports.

[99] Ma S., Sun S., Geng L., Song M., Wang W., Ye Y. (2020). Caloric restriction reprograms the single-cell transcriptional landscape of Rattus norvegicus aging. Cell.

[100] Abella V., Scotece M., Conde J., Gómez R., Lois A., Pino J. (2015). The potential of lipocalin-2/NGAL as biomarker for inflammatory and metabolic diseases. Biomarkers.

[101] Shashidharamurthy R., Machiah D., Aitken J.D., Putty K., Srinivasan G., Chassaing B. (2013). Differential role of lipocalin 2 during immune complex–mediated acute and chronic inflammation in mice. Arthritis & Rheumatism.

[102] Guo H., Jin D., Chen X. (2014). Lipocalin 2 is a regulator of macrophage polarization and NF-κB/STAT3 pathway activation. Molecular Endocrinology.

[103] Zhang Y., Foncea R., Deis J.A., Guo H., Bernlohr D.A., Chen X. (2014). Lipocalin 2 expression and secretion is highly regulated by metabolic stress, cytokines, and nutrients in adipocytes. PloS One.

