# Supplementary material for: Differential regulation of the immune system in a brain-liver-fats organ network during short-term fasting
Source: Mol Metab. 2020 Jun 8;40:101038. doi: 10.1016/j.molmet.2020.101038 (PMC7339127; doi:10.1016/j.molmet.2020.101038)
Supplement: Multimedia component 1 [file mmc1.docx]

**SUPPLEMENTARY INFORMATION**

**Additional file 1: Figure S1-S10.**

**Additional file 2: Table S1.** Gene expression estimates as normalized counts (NCs) for all filtered genes and all samples.

**Additional file 3: Table S2.** Phase cluster analysis results based on the high variable genes (HVGs).

**Additional file 4: Table S3.** Differential expression analysis for all pairwise Phase comparisons for OB, BRN, CBL, BST, STM, LIV, iBAT, and psWAT.

**Additional file 5: Table S4.** Gene expression estimates as a log_2_ (x+1) normalized counts (NCs) for the filtered DEGs for all samples. Median, mean and SEM are also displayed.

**Additional file 6: Table S5.** Reduced gene ontology (GO) enrichment terms for all subcDEGs in all organs.

**Additional file 7: Table S6.** The overlapping enriched gene ontology (GO) terms for all subcDEGs that were used for the *brain-liver-fats* organ network analysis.

**Additional file 8: Table S7.** Enriched categories for the Reactome pathway enrichment of the *brain-liver-fats* organ network.

**Additional file 9: Table S8.** Gene list compiled from 52 triaged PubMed articles, using Gene Retriever™.
