# Supplementary material for: Differential regulation of the immune system in a brain-liver-fats organ network during short-term fasting
Source: Mol Metab. 2020 Jun 8;40:101038. doi: 10.1016/j.molmet.2020.101038 (PMC7339127; doi:10.1016/j.molmet.2020.101038)
Supplement: Multimedia component 10 [file mmc10.docx]

**Differential regulation of the immune system in a *brain-liver-fats* organ network during short term fasting**

Susie S.Y. Huang^1,#,^*; Melanie Makhlouf^1,#^; Eman H. AbouMoussa^1^; Mayra L. Ruiz Tejada Segura^2,3,4^; Lisa S. Mathew^1^; Kun Wang^1^; Man C. Leung^1^; Damien Chaussabel^1^; Darren W. Logan^5^; Antonio Scialdone^2,3,4^; Mathieu Garand^1,†^; Luis R. Saraiva^1,6, †,^*

^1^ Sidra Medicine, PO Box 26999, Doha, Qatar.

^2^ Institute of Epigenetics and Stem Cells, Helmholtz Zentrum München, Marchioninistraße 25, 81377 München, Germany

^3^ Institute of Functional Epigenetics, Helmholtz Zentrum München, Ingolstädter Landstraße 1, 85764 Neuherberg, Germany

^4^ Institute of Computational Biology, Helmholtz Zentrum München, Ingolstädter Landstraße 1, 85764 Neuherberg, Germany

^5^ Wellcome Sanger Institute, Wellcome Genome Campus, Hinxton, Cambridge, CB10 1SD, UK.

^6^ Monell Chemical Senses Center, 3500 Market Street, Philadelphia, PA 19104, USA.

^#^ equal contribution (co-first)

^†^ equal contribution (co-senior)

* Correspondence and requests for materials should be addressed to S.S.Y.H (susie.s.y.huang@gmail.com) or L.R.S. (email: saraivalmr@gmail.com, twitter: @saraivalab)

**Additional file 1: Supplementary Figures S1-S10**


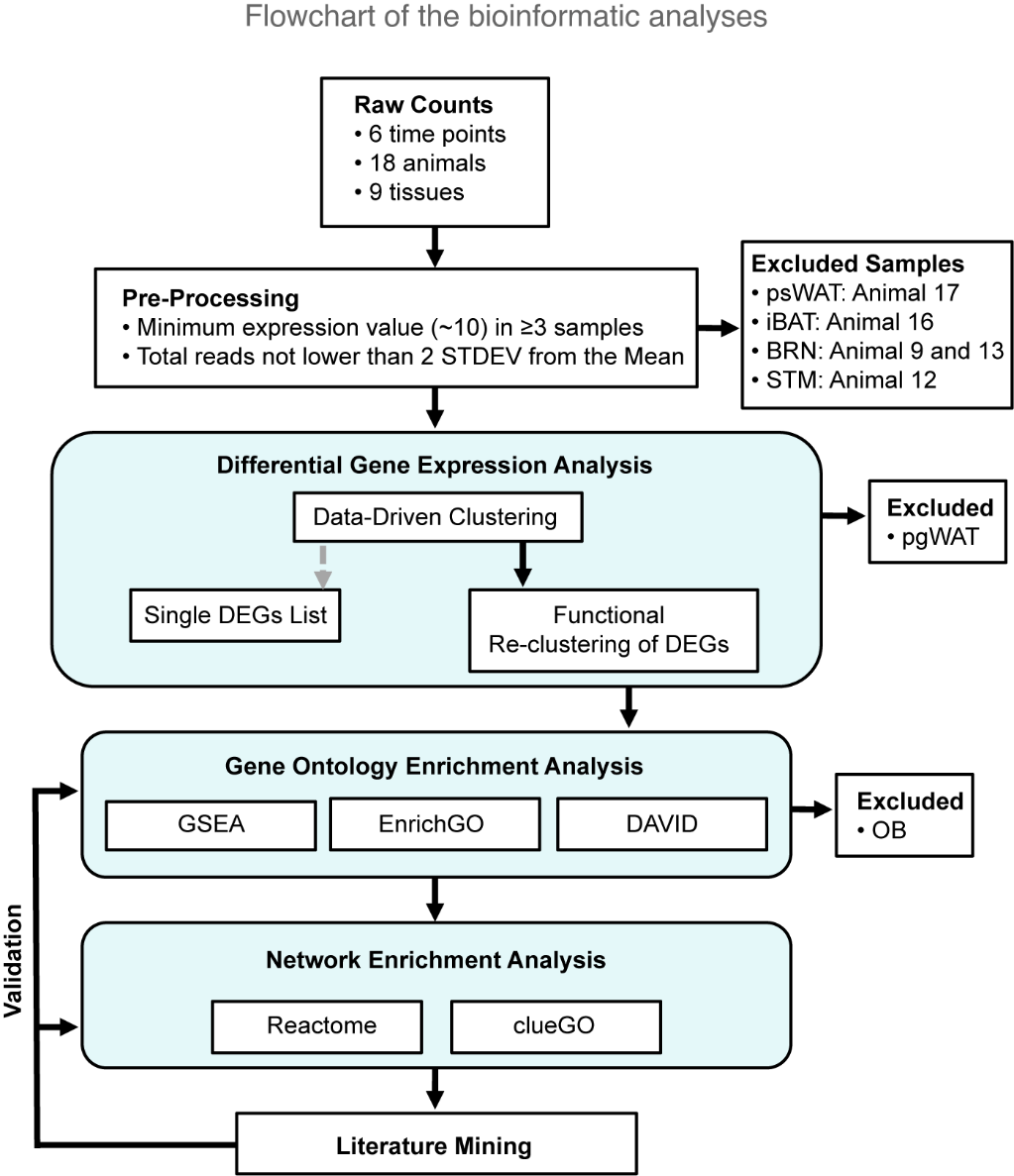


**Additional file 1: Figure S1. Schematic of the bioinformatic workflow.**

Genes and samples were filtered for minimum expression threshold, replicate numbers, and outlier exclusion. A data-driven approach was then used to group samples into Phases to derive the differentially expressed genes which were then functionally clustered by their semantic similarities prior to gene ontology analysis. A group of four highly overlapping organs (BRN, LIV, iBAT and psWAT) was carried forward to protein enrichment analyses and the enriched processes were validated using literature mining.


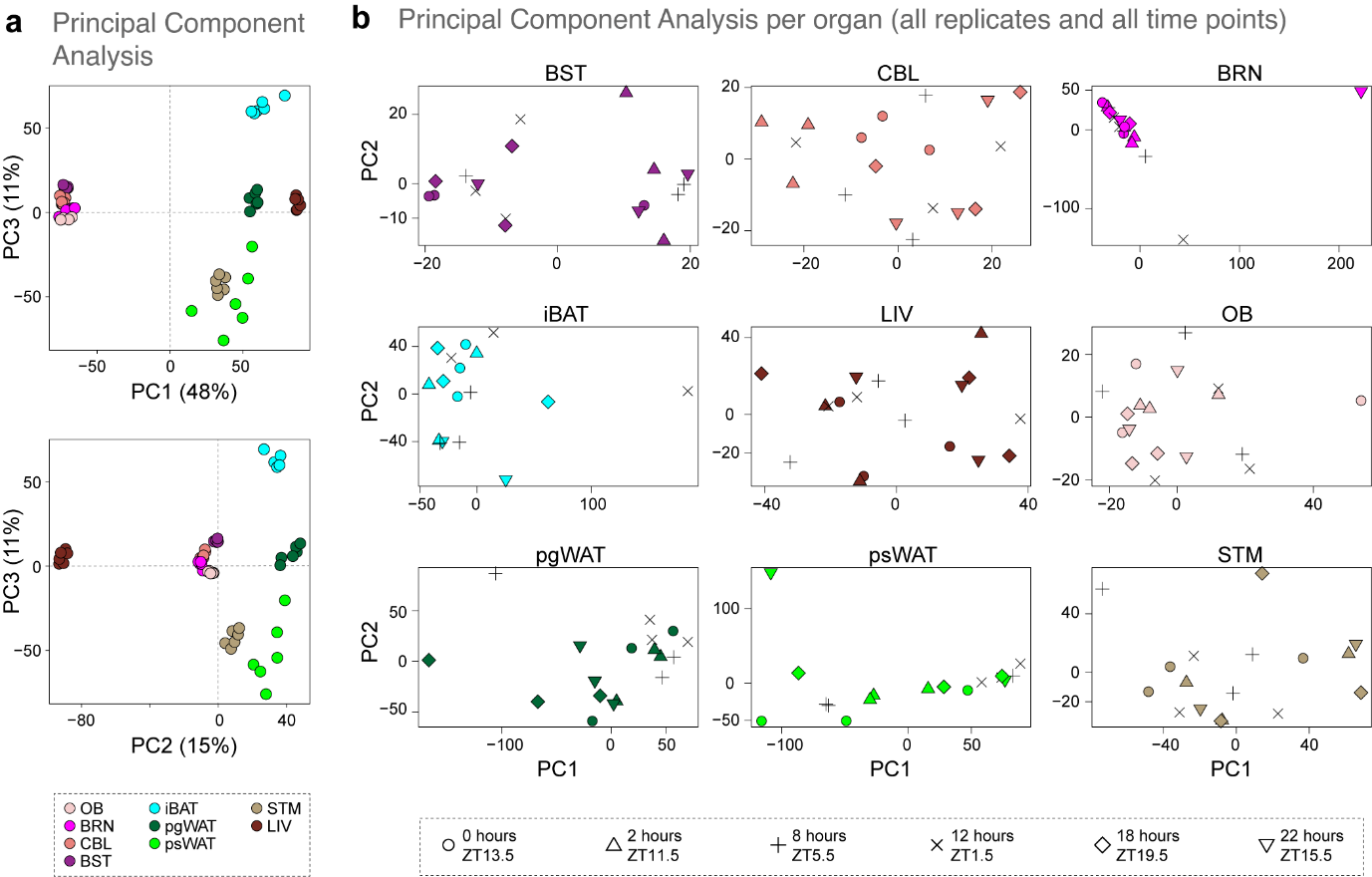


**Additional file 1: Figure S2. Principal component analysis of all organs across all fasting times.**

**a)** PCA analysis of PC1 vs PC3 (top) and PC2 vs PC3 (bottom) of the 9420 expressed genes in all samples. Each dot represents the gene expression profile of an organ (indicated by the colour) at a specific time point. Percentages of the variance explained by the PCs are indicated in parentheses.

**b)** PCA analysis of PC1 vs PC2 of the non-zero expressed genes in the nine different organs. Each symbol represents the overall gene expression of a sample at a specific time point

Organ abbreviations: OB - olfactory bulb, BRN - brain, CBL - cerebellum, BST - brainstem, STM - stomach, LIV - liver, iBAT - interscapular brown adipose tissue, pgWAT - perigonadal white adipose tissue, and psWAT - posterior-subcutaneous white adipose tissue.


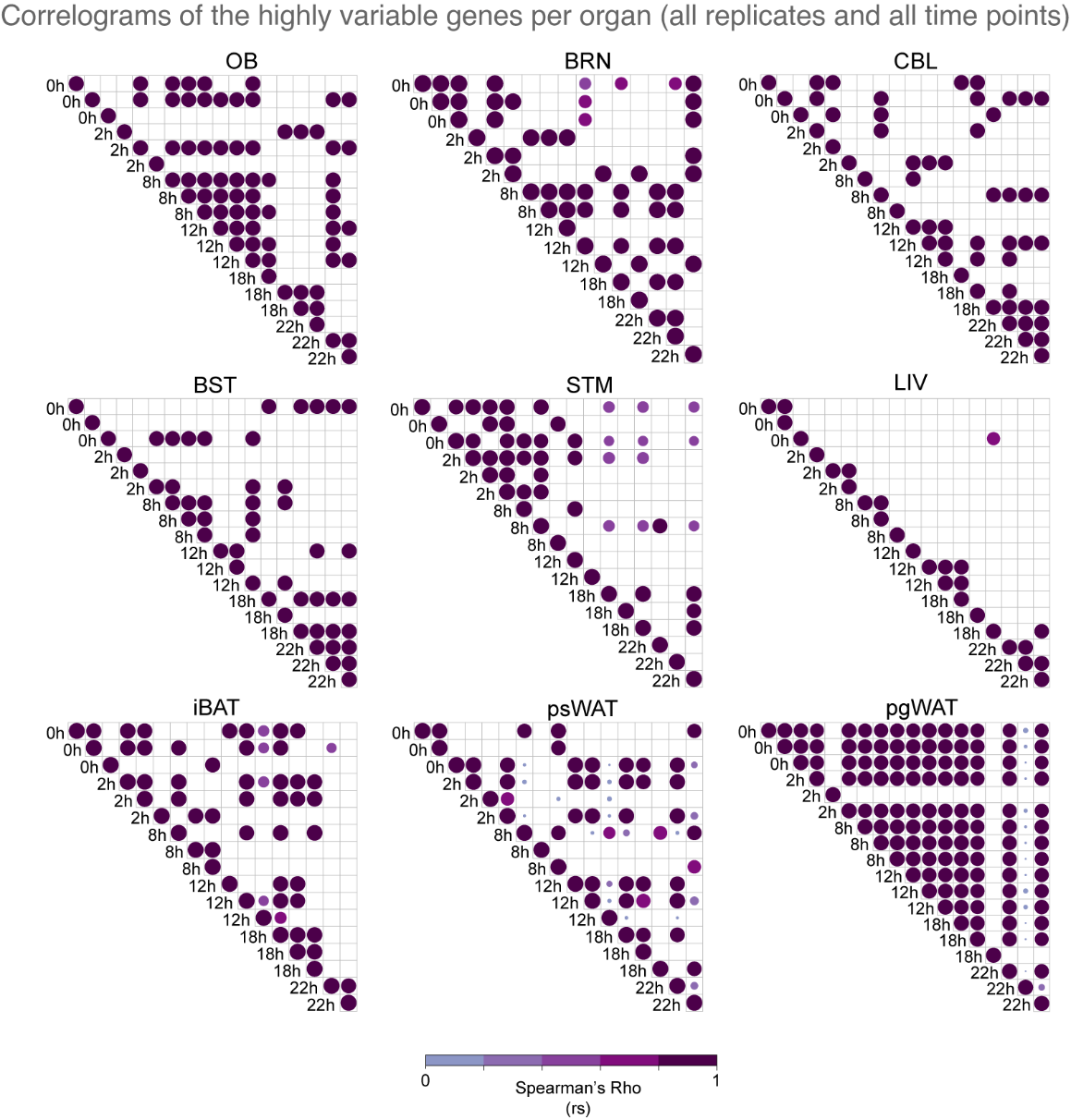


**Additional file 1: Figure S3. Spearman’s correlation matrix of the expressions of the highly variable genes among each sample in the nine organs.**

In the correlogram, only significant (p.adusjst < 0.05) correlations are plotted, and the circle size and color indicate the magnitude and direction of the correlation (Spearman rho, rs).

Organ abbreviations: OB - olfactory bulb, BRN - brain, CBL - cerebellum, BST - brainstem, STM - stomach, LIV - liver, iBAT - interscapular brown adipose tissue, pgWAT - perigonadal white adipose tissue, and psWAT - posterior-subcutaneous white adipose tissue.


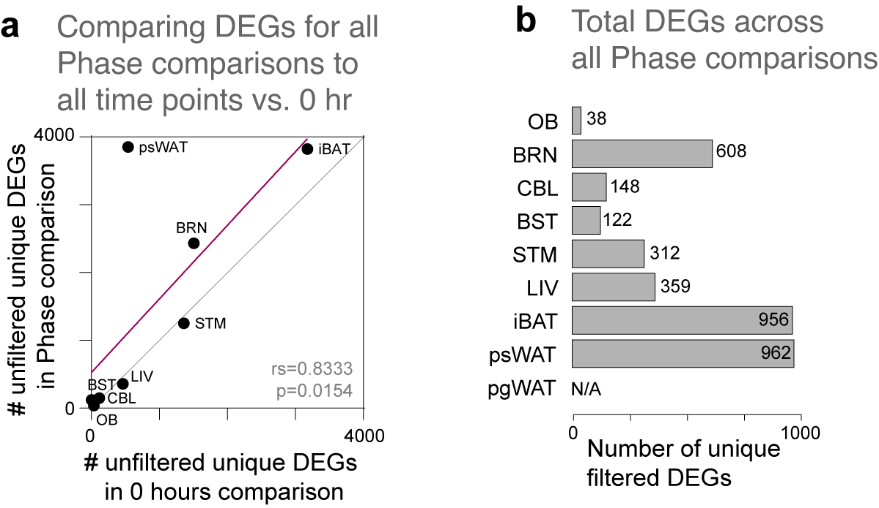


**Additional file 1: Figure S4. Differential expression analysis.**

**a)** A comparison between the differentially expressed genes (DEGs) determined in a pairwise manner between the Phases (y-axis) and between each time point against zero (x-axis) yielded similar numbers of DEGs. Spearman’s correlation coefficients (rs) and associated p-values (p) are noted on the bottom right of the graph. The purple line represents the linear regression.

**b)** Number of the filtered DEGs from the all pairwise fasting Phase comparisons in all organs that were used in the downstream analyses (see Methods). Since samples from pgWAT did not split into fasting Phases, no DEGs for the organ were available (N/A).


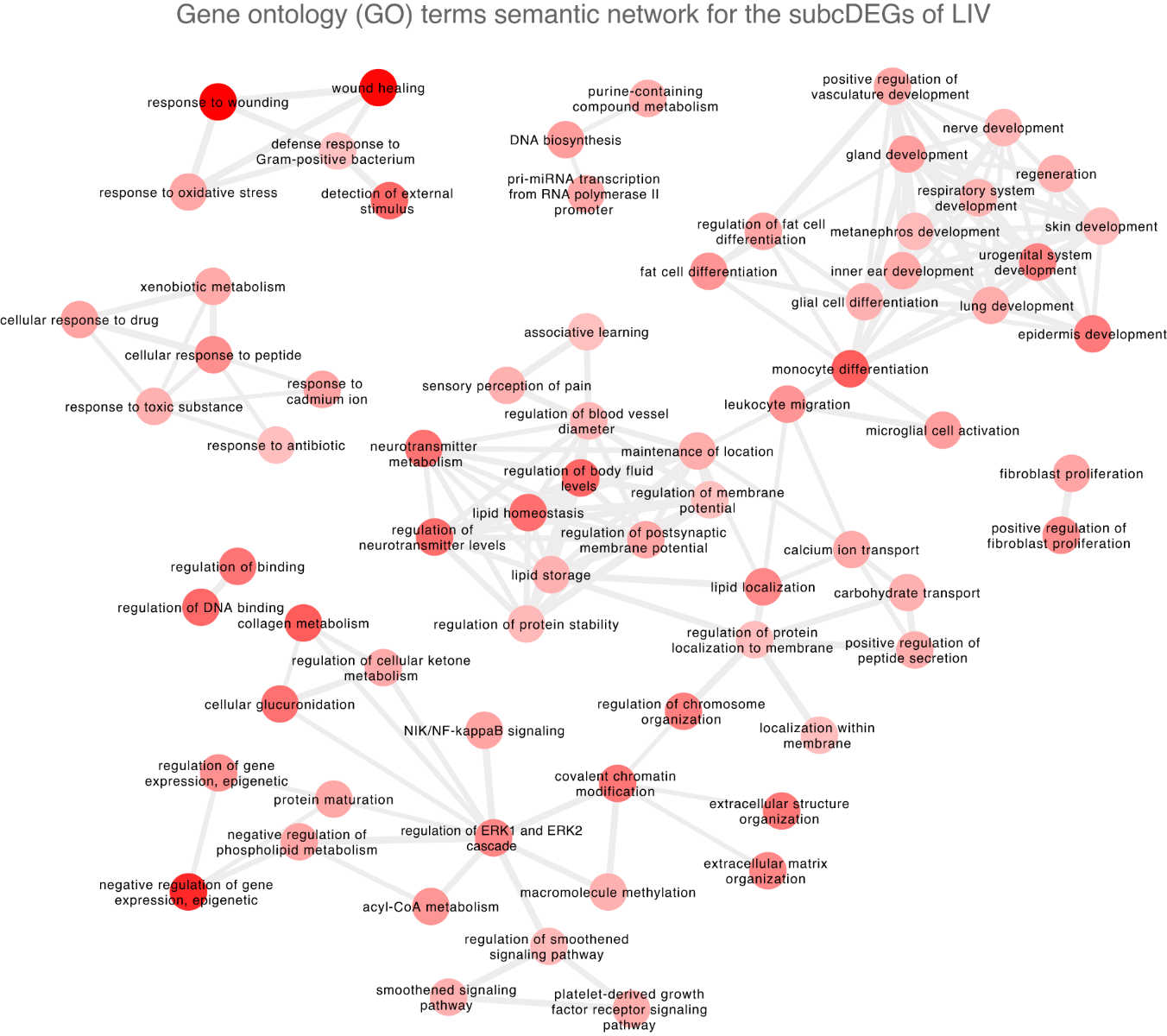


**Additional file 1: Figure S5. Semantic network of the gene ontology (GO) terms enriched in the liver.**  Nodes are GO terms and the intensity of the colour indicates enrichment (-log_10_ p-value, darker shade indicates lower p-values). Highly similar GO terms are linked by edges, where the line width indicates the degree of similarity.


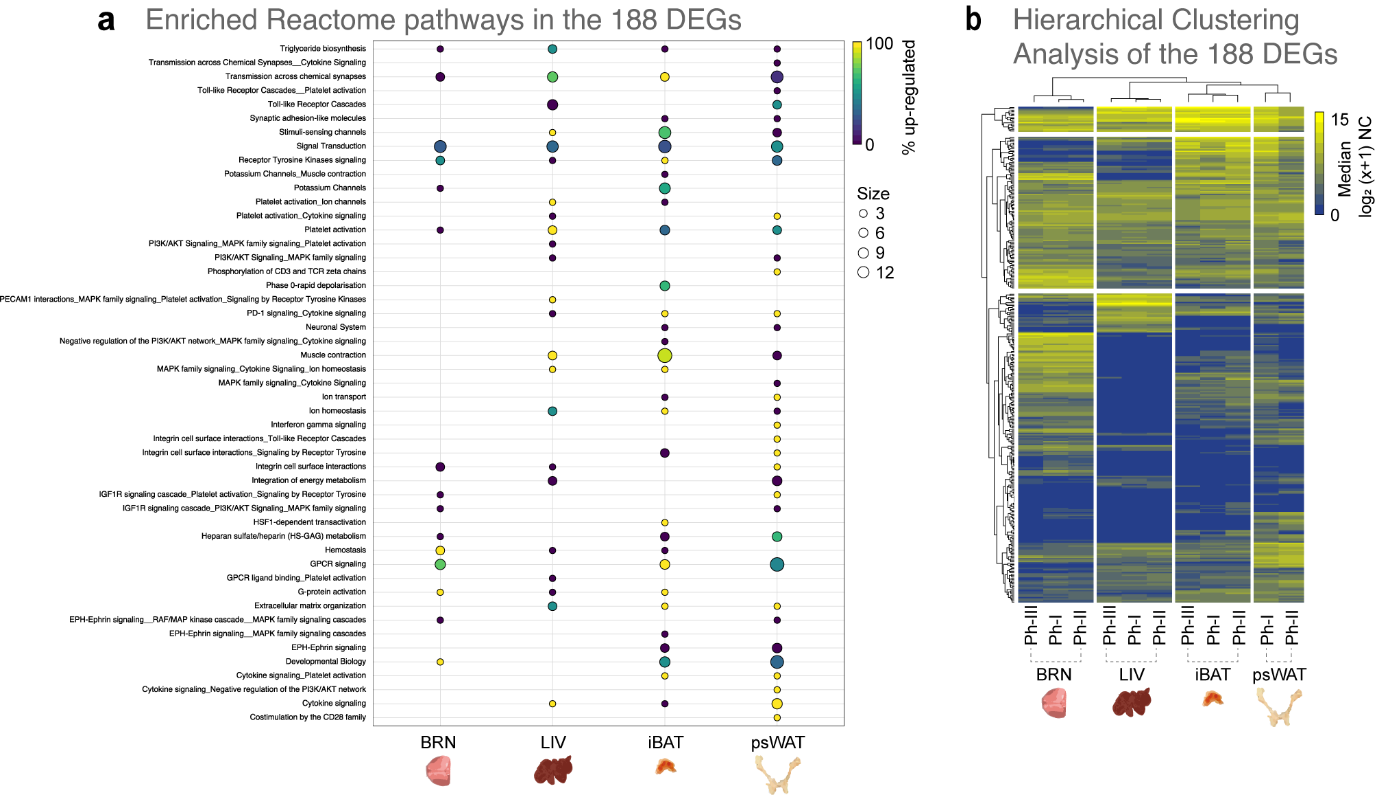


**Additional file 1: Figure S6. Gene expression of the *brain-liver-fats* organ network.**

**a)** Percent of genes up-regulated in the summarized enriched Reactome terms of the *brain-liver-fats* organ network (Fig. 4a) in BRN, LIV, iBAT, and psWAT. Similar/redundant Reactome terms were combined for simplicity of representation.

**b)** Hierarchical clustering analysis of the 188 genes from the *brain-liver-fats* organ network. Median mRNA expression levels are represented on a log_2_ (x+1) scale of normalized counts (NC) (0 - not expressed; 15 - highly expressed) per fasting Phases (Ph) and across the four organs.

Organ abbreviations: BRN - brain, LIV - liver, iBAT - interscapular brown adipose tissue, and psWAT - posterior-subcutaneous white adipose tissue.


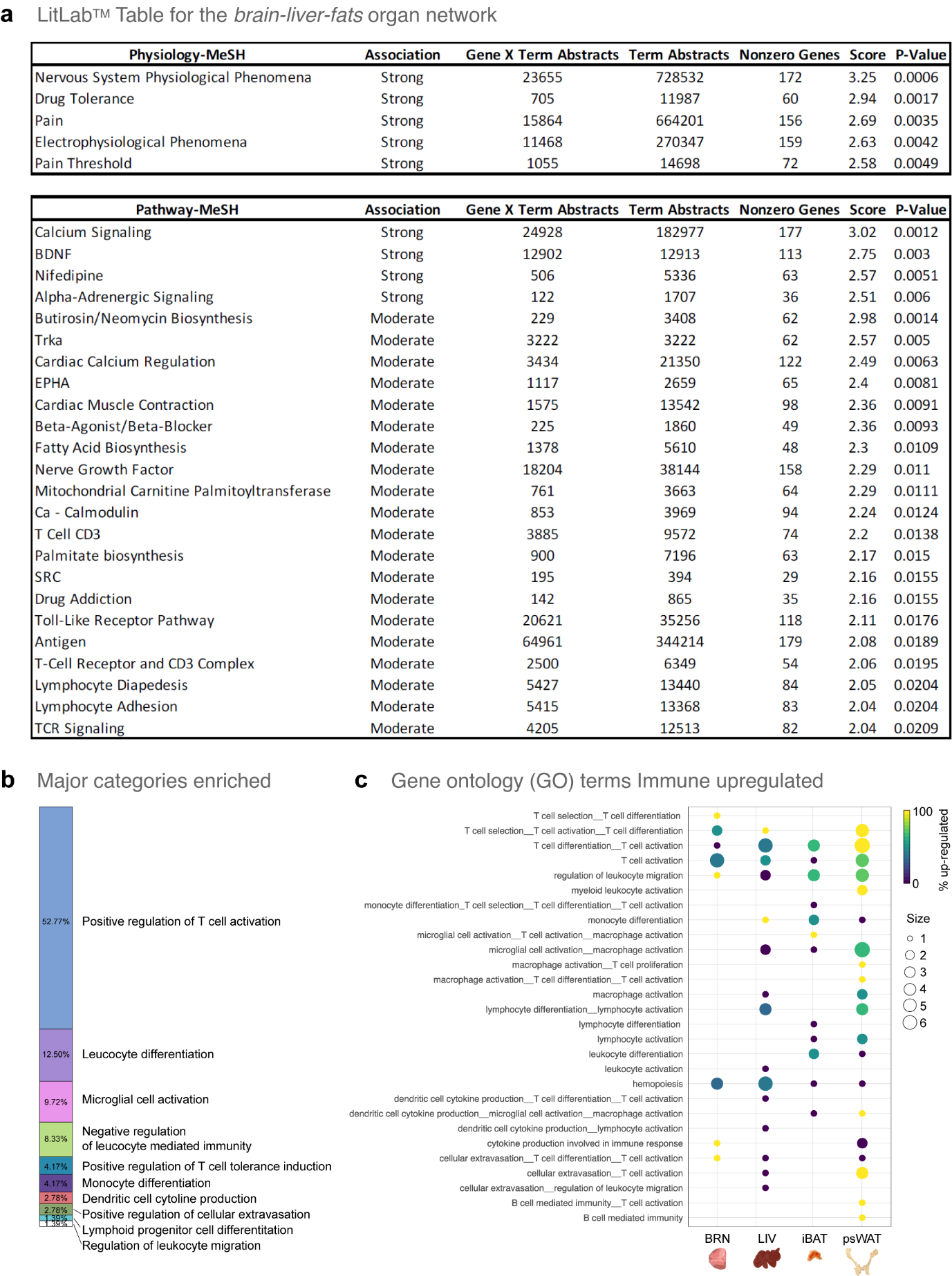


**Additional file 1: Figure S7. Short-term fasting modulates immune processes in the *brain-liver-fats* organ network.**

**a)** Summary of the significant Physiology and Pathway-specific Medical Subject Headings (MeSHs) associated with the 188 organ network genes resulting from the association analysis performed using LitLab™.

**b)** The representative categories and percentages of the immune-specific GO terms enriched from the 96 genes extracted from the 37 overlapping GO terms from BRN, LIV, iBAT and psWAT that passed the significant threshold set.

**c)** Proportion of the up-regulated gene from the 96 genes from the organ network that resulted in a significant immune-specific GO network enrichment.

Organ abbreviations: BRN - brain, LIV - liver, iBAT - interscapular brown adipose tissue, and psWAT - posterior-subcutaneous white adipose tissue.


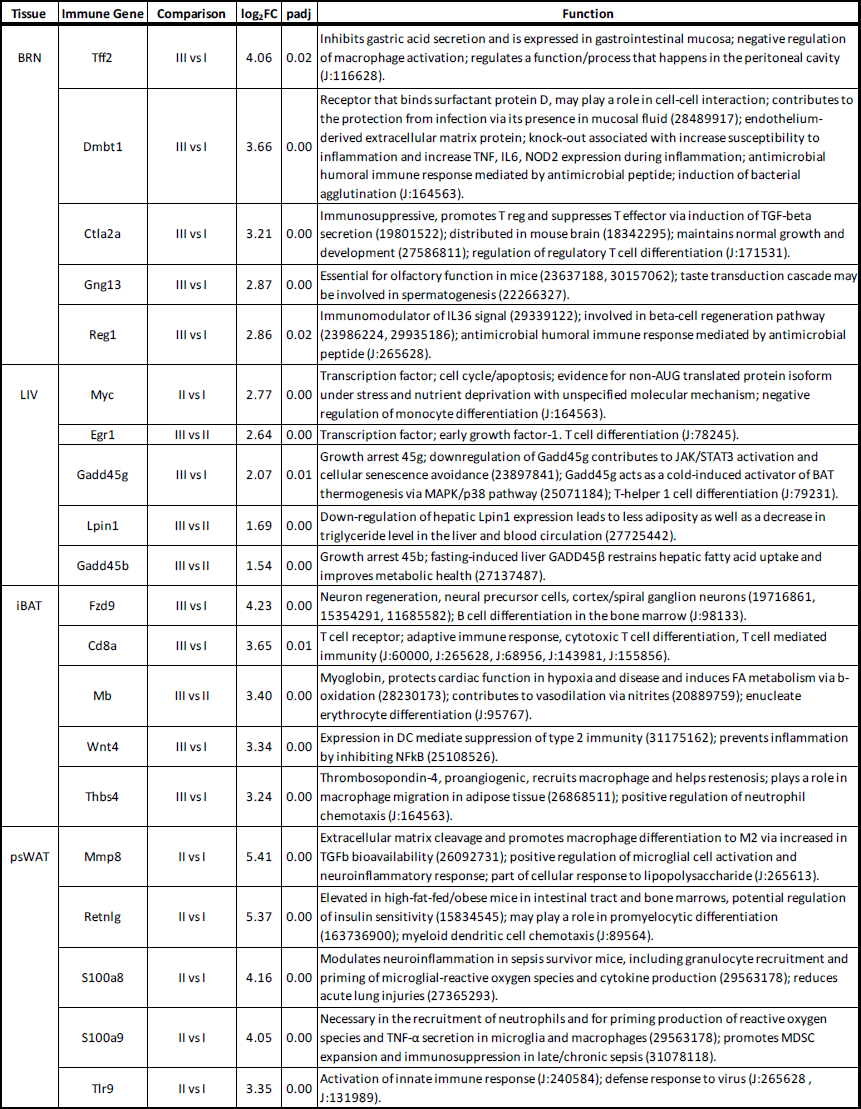


**Additional file 1: Figure S8. Immune-related DEGs in BRN, LIV, iBAT and psWAT.**

Top five up-regulated immune-related genes among DEGs of the four organs (BRN, LIV, iBAT and psWAT) and their literature-supported functions. The immune-related functions of each gene were extracted from NCBI and MGI databases, as indicated by their PubMed IDs and reference IDs.


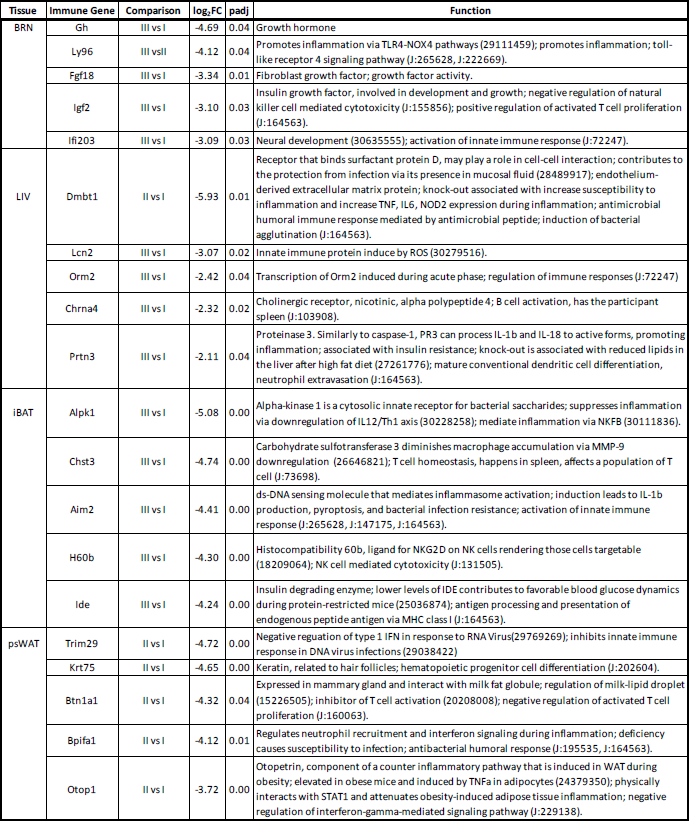


**Additional file 1: Figure S9. Immune-related DEGs in BRN, LIV, iBAT and psWAT.**

Top five down-regulated immune-related genes among DEGs of the four organs (BRN, LIV, iBAT and psWAT) and their literature-supported functions. The immune-related functions of each gene were extracted from NCBI and MGI databases, as indicated by their PubMed IDs and reference IDs.


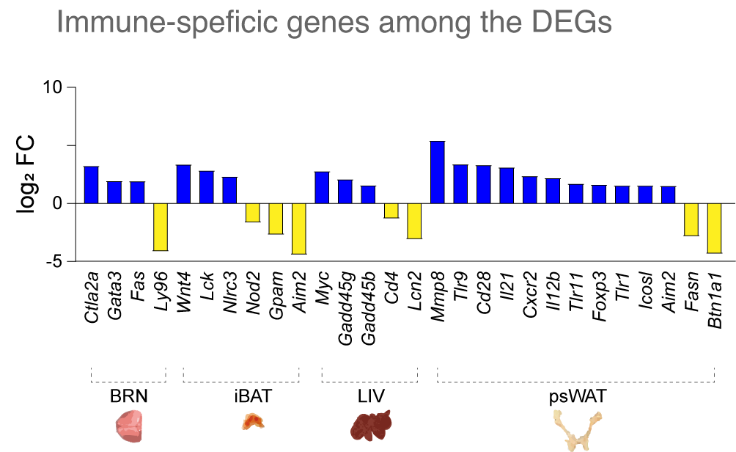


**Additional file 1: Figure S10. Log_2_FC of select immune genes in the *brain-liver-fats* organ network in response to fasting time.** Yellow and blue bars depict genes with decreased and increased expression with fasting time, respectively.

Organ abbreviations: BRN - brain, LIV - liver, iBAT - interscapular brown adipose tissue, and psWAT - posterior-subcutaneous white adipose tissue.
